# Motor features that distinguish isolated REM sleep behavior disorder patients from healthy controls: A systematic review

**DOI:** 10.1177/1877718X251359225

**Published:** 2025-09-01

**Authors:** Salma Elasfar, Hajr Hameed, Kaylena Ehgoetz Martens

**Affiliations:** Department of Kinesiology and Health Sciences, University of Waterloo, Waterloo, Ontario, Canada

**Keywords:** rapid-eye-movement sleep behavior disorder, parkinson's disease, dementia with Lewy bodies, mobility, gait

## Abstract

Individuals with isolated REM sleep behavior disorder (iRBD) are at high risk of developing α-synucleinopathies, particularly Parkinson's disease (PD) and dementia with Lewy bodies (DLB). With the development of potential neuroprotective treatments for synucleinopathies, including PD, identifying clinical features that can allow for tracking subtle changes in prodromal disease and thereby monitoring risk of phenoconversion in iRBD is paramount. Subtle motor deficits have been suggested to be present in iRBD, making them potentially important clinical markers for predicting future phenoconversion. This review aims to summarize existing literature that has investigated differences in motor function between iRBD and healthy individuals, as well as progression of motor decline in iRBD. 39 eligible studies were included in this review. The results suggest that quantitative motor assessments may be more sensitive to motor impairments in this population than clinical scales. Moreover, dual-tasking tended to unmask subtle motor deficits in individuals with iRBD, particularly in gait, balance, and tapping assessments. Longitudinal studies demonstrate that motor function worsens over time in iRBD, with earliest signs of motor deficits and clear progression in tapping assessments in particular. Larger longitudinal studies that use quantitative methods of motor assessments are needed to better characterize motor progression in iRBD, and confirm the reliability of different motor markers for predicting phenoconversion of iRBD into PD and other synucleinopathies.

## Plain language summary

People with isolated REM sleep behavior disorder (iRBD) have a higher risk of developing Parkinson's disease (PD) and dementia with Lewy bodies (DLB). Currently, researchers are investigating different treatments to protect against the risk of developing these diseases in those with iRBD. To do this, it is important to identify markers that can help clinicians monitor progression of iRBD and assess the risk of developing PD or DLB. Studies have shown that individuals with iRBD exhibit subtle motor deficits before developing PD or DLB, which could suggest changes in motor function may be important signs to predict disease progression. This review aims to summarize existing research on motor deficits in iRBD compared to healthy individuals, and how motor function changes over time in iRBD. We found 39 relevant studies and included them in this review. Our findings suggest that tests that measure motor skills precisely, for example, with wearable sensors or motion capture camera, are better at capturing motor deficits in iRBD compared to tests commonly used in clinics. We also found that performing motor tests by adding a secondary task, for example, counting backwards by sevens while walking, revealed motor deficits in iRBD, particularly during walking, balance and finger and keyboard tapping tasks. The longitudinal studies in this review showcased that motor function declines over time in iRBD, but some of the earliest changes in motor function are in tapping assessments. Future research should involve larger study samples with longitudinal follow-up to improve our understanding of how motor function changes over time in iRBD and confirm whether these motor features can predict conversion of iRBD into PD and DLB.

## Introduction

Isolated REM sleep behavior disorder (iRBD) is a parasomnia characterized by a loss of muscle atonia during REM sleep and dream enactment, with an estimated prevalence ranging from 0.5% to 2.01% in the general population^1–3^. Polysomnography (PSG) is currently the gold standard for identifying iRBD.^4^ Individuals with iRBD have a high risk of developing α-synucleinopathies, particularly Parkinson's disease (PD) and dementia with Lewy bodies (DLB).^5,6^ The estimated risk of phenoconversion is 33.5% after 5 years, 82.4% after 10 years, and 96.6% after 14 years.^6^

For this reason, iRBD represents an important target for potential neuroprotective therapies, and an important model for understanding disease progression of prodromal PD.^7^ Therefore, it is of paramount importance to establish biomarkers that can reliably identify and monitor subjects at high risk of rapid phenoconversion to PD. Imaging studies have demonstrated decreased striatal dopamine transporter uptake and substantia nigra hyperechogenicity as potential markers to identify individuals with iRBD at risk of phenoconversion within three years.^8^ However, these imaging methods are relatively expensive and time-consuming, and may not be readily available in all healthcare settings.^4^

Subtle motor deficits have been reported to be present in iRBD, and therefore, may be helpful markers for predicting future phenoconversion. Therefore, motor assessment methods may be relatively inexpensive tools that can aid in screening for iRBD and monitoring disease progression, particularly risk of phenoconversion. Several studies have aimed to characterize the motor impairment seen in iRBD using various motor assessment methods, however, to our knowledge, no review has aimed to summarize the existing evidence.

Therefore, the purpose of this systematic review was to a) understand what motor features best distinguish individuals with iRBD from healthy controls (HCs), and b) explore how motor features progress over time in iRBD compared to HCs.

## Methods

This systematic review protocol was a priori published on PROSPERO (registration number: CRD42024509669) and guided by the Preferred Reporting Items for Systematic Reviews and Meta-Analyses (PRISMA) statement and checklist.^9^ The protocol has deviated from the a priori published protocol, specifically with regard to the inclusion criteria, as we have included studies that do not include healthy controls if they have a longitudinal design that measures motor function in participants with iRBD over time. This was done to appropriately address our second aim.

### Search strategy and study selection

A detailed systematic search of the existing literature was performed in PubMed, Scopus, EMBASE, and CINAHL between February 2024 and April 2024. Keywords and Medical Subject Headings used to search the databases are listed in Table 1. There was no date limitation. All articles were imported into Covidence, an online systematic review software, and duplicates were removed.^10^

**Table 1.** Keywords and MeSH headings (*) used in the search strategy.

Key words for motor function	Key words for isolated RBD
Mobility	Rapid eye movement sleep behavior disorder
Mobility limitation(*)	Rapid eye movement sleep behaviour disorder
Motor behavior	REM sleep behavior disorder(*)
Motor skills(*)	REM sleep behaviour disorder
Motor disorder(*)	RBD
Motor performance	iRBD
Locomotion(*)	
Ambulation	
Gait(*)	
Gait analysis(*)	
Gait disorders(*)	
Walking(*)	
Walking speed(*)	
Walk	
Step	
Stride	
Cadence	
Postural control	
Balance	
Postural balance(*)	
Kinematics	
Falls	
Accidental falls(*)	

Two independent reviewers (SE and HH) conducted the process of study selection, starting with title and abstract screening to identify relevant studies, followed by full text review of relevant studies to identify studies that met the following eligibility criteria:

- Study was conducted with human participants.
- Study includes participants with confirmed or probable isolated REM sleep behavior disorder, without PD or other neurodegenerative conditions.
- Study includes a healthy control group (for cross-sectional studies only). Longitudinal studies without a control group that follow individuals with RBD over time were included.
- Study includes a clearly described motor assessment and clearly defined motor outcomes.

Studies were excluded if they were not written in English or not published in peer-reviewed journals. Reviews, case studies, case series, conference proceedings, books, and dissertations were also excluded. Disagreement during the screening process was resolved by another reviewer (KEM). Finally, reference lists of relevant studies that met the eligibility criteria were manually screened to identify articles that were not captured in the electronic database searches.

### Data extraction

Data extraction was performed in Covidence. The following information was extracted from eligible studies by SE and HH: authors, country, date of publication, journal of publication, inclusion and exclusion criteria, methods for diagnosing iRBD, sample size (% Female), mean age, follow-up duration (if applicable), cognitive test scores, motor assessment method(s), motor assessment outcome(s), and covariate adjustments. We additionally extracted mean and standard deviation (or median and range if mean and standard deviation were not reported) of the motor outcomes, as well as the results of the statistical analyses (e.g., p-values for group comparisons, or AUC, sensitivity, and specificity for discriminatory analyses). Data extracted were compared between both reviewers to ensure accuracy. Any disagreements were resolved by checking the study for the correct data.

### Risk of bias/quality assessment

A quality assessment of each eligible study was conducted independently by two reviewers (SE and HH) using a modified Downs and Black checklist, which assesses reporting, external validity, internal validity, and power.^11^ This form of risk of bias assessment has been previously used in systematic reviews focusing on motor assessments in various populations, including PD.^12–15^

The checklist was modified to exclude items that applied to intervention studies, and only items relevant for observational studies were used.^11^ The items included were numbered 1, 2, 3, 5, 6, 7, 10, 11, 12, 16, 18, 20, 21, 22, 25, and 27, making the maximum possible score 16. Item 5 was modified so that principal confounders included age, gender, and cognitive function, which can impact motor behavior. Item 17 was also used for studies with longitudinal follow-up.^11^ Items 17, 21, and 22 were not applicable to longitudinal studies without a control group. Item 25 was assessed in terms of statistical analyses for the motor function outcomes only, as this is the focus of our review. Disagreements were resolved through consensus between the two reviewers and inter-rater reliability was calculated. Scores were converted into percentages, and the quality of each study was classified as high (>66.8%), medium (33.4–66.7%) or low (<33.3%).

## Results

### Search strategy and study selection results

**Figure 1.**
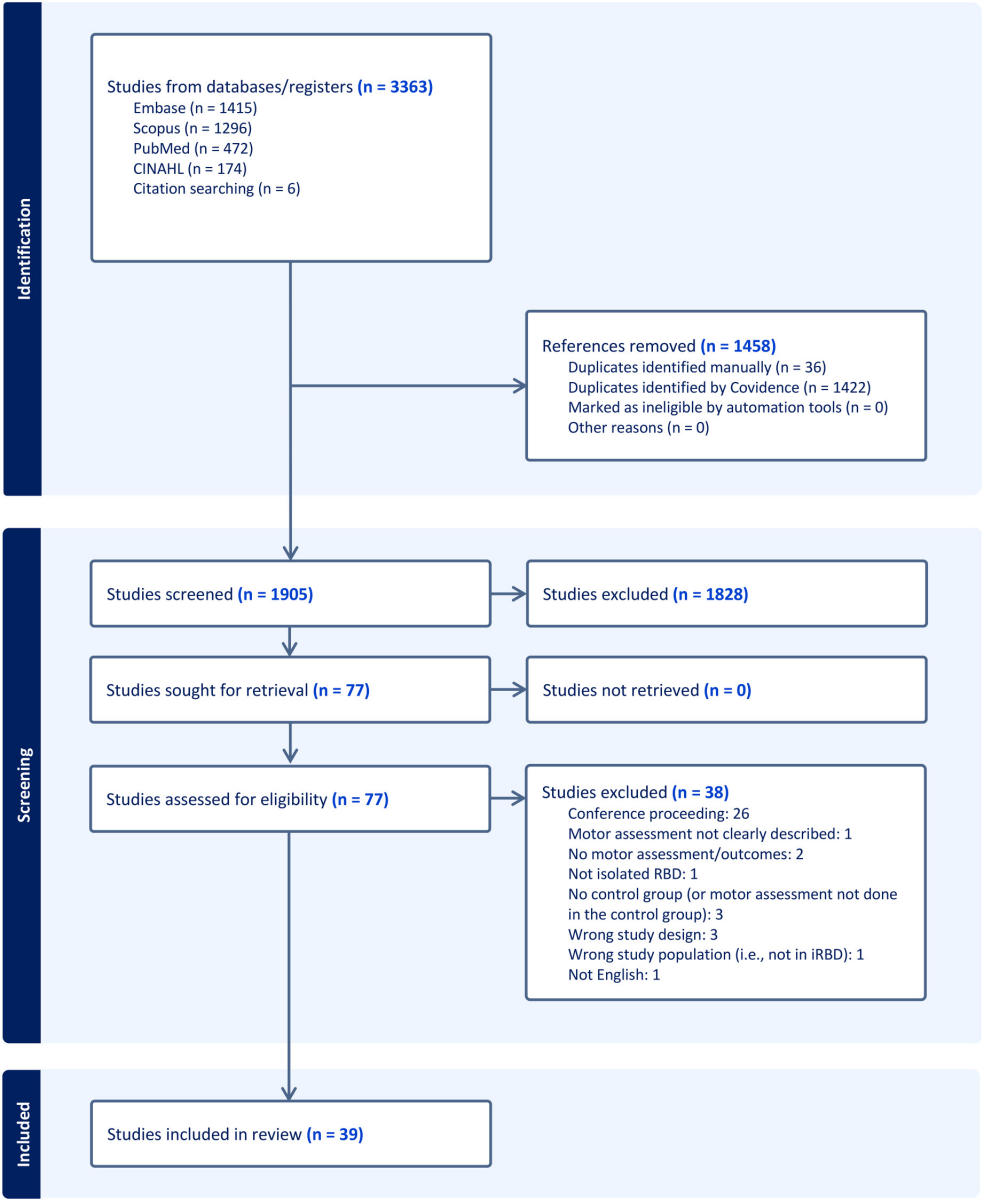
Flow diagram of search strategy and study selection results.

The results of the study selection process are summarized in Figure 1. Our electronic database search resulted in a total of 3363 articles. After duplicates were removed, 1905 studies remained for title and abstract screening. From these, we identified 77 relevant studies for full text review, five of which were identified from manual citation searching. After applying the eligibility criteria, 38 studies were excluded: 26 were excluded because they were conference abstracts (four of which reported on preliminary results from longitudinal studies), two were excluded for not including the results of their motor assessments, one for not clearly describing their motor assessment, and three for conducting the motor assessments in the iRBD group only, not the control group. Three studies had the wrong study design/aims for our review, for example, two compared iRBD groups that have converted to DLB or PD, while another subdivided and compared the iRBD group by cognitive performance. Two studies were excluded for the wrong patient population (one was a study in PD, and one recruited participants that did not have isolated RBD). Finally, one study was excluded as it was not written in English. Therefore, we identified 39 studies that were included in this review.^16–54^

### Cross-sectional results

All studies included in this review are summarized in Table 2 in terms of authors, year of publication, country, inclusion/exclusion criteria, method of iRBD diagnosis, motor assessment methods and outcomes, and main findings. Table 2 summarizes the results of all cross-sectional studies by motor assessments and whether group differences between iRBD and HCs were found or not. We have also included supplemental tables, which summarize study results by the type of motor assessment conducted.

**Table 2.** Full summaries for included studies.

Authors, Year, Country	Inclusion/Exclusion Criteria	Characteristics of Participants with iRBD	Healthy Participants Characteristics	Other participant groups	Motor assessment(s) and outcomes	Follow-up	Covariates	Statistical Method(s) of Interest	Main study findings
**Cross-sectional**									
Alibiglou et al., 2016, United States of America^16^	**Inclusion criteria**: Ability to ambulate independently without an assistive device. for iRBD group: PSG-confirmed RBD (ICSD-II criteria). **Exclusion criteria**: Diagnosis of idiopathic PD, Hoehn & Yahr stage 2 to 3, another neurological disorder, a tremor score of greater than 2 on items 20 and 21 of the UPDRS, a history of neurosurgery, a Mini Mental State Examination score of less than 26, any musculoskeletal disorder that affected walking	n = 10 (40% F), mean age = 61.5 ± 8.6; disease duration = 1.34 ± 0.94; MMSE = 30 ± 0	n = 10 (20% F), mean age = 62.7 ± 11.5; MMSE = 30 ± 0	PD without FOG: n = 10 (20% F); mean age = 61.05 ± 9.4; MMSE = 30 ± 1 PD with FOG: n = 10 (20% F); age = 65.9 ± 11.2; MMSE = 30 ± 1	UPDRS-III total score Self-initiated, forward-stepping task: Participants stand stationary on 2 adjacent force platforms (464 × 508 mm; AMTI, Watertown, Massachusetts), instructed to wait 3–5 s then take at least 3 steps forward with their right or left leg. Loading GRF and unloading GRF and peak CoP excursions (CoP-Right, CoP Post-Peak_1_,and CoP Post-Peak_2_) recorded. 10 trials per leg collected. EMG (Bagnoli-16 System) recorded bilaterally from TA. Incidence of trials with no anticipatory postural adjustments recorded.	N/A	None; participants were matched by age and sex; MMSE did not differ between groups	ANOVAs, partial correlations	UPDRS-III was not different between iRBD and HCs. The iRBD group exhibited significant reductions in the posterior shift of CoP during the propulsive phase of gait initiation and in the duration of the initial EMG burst in TA in participants with iRBD. This was comparable to the deficits in gait initiation observed in a PD group with FOG.
Arora et al, 2018, United Kingdom^17^	**Inclusion criteria**: for iRBD group: PSG-confirmed RBD (ICSD-III criteria)	n = 104 (12% F); mean age = 64.5 ± 9.4; MMSE = 26.9 ± 4.2; MoCA = 25.5 ± 2.6	n = 84 (33% F); mean age = 66.3 ± 9.1; MMSE = 28.5 ± 1.5; MoCA = 26.9 ± 2.2	PD: n = 334 (37% F); mean age = 66.1 ± 9.0; MMSE = 27.6 ± 3.0; MoCA = 25.2 ± 3.7	Finger tapping task: Tap screen alternately keeping regular rhythm. Reaction time task: Press and hold on-screen button as soon as it appears and release it as soon as it disappears. Balance task: Stand upright unaided for 30 s. Gait task: Walk 20 steps forward, turn around and return back to starting position. Rest tremor task: Sit upright, hold the phone in your tremor dominant hand and rest it lightly in your lap, and close your eyes and count backward from 100. Postural tremor: Sit upright and hold the phone in your tremor dominant hand, with the arm outstretched in front of you.	N/A	None; iRBD group was younger than controls and PD groups, and more likely to be male.	Statistical machine learning methods (random forests)	Smartphone tests distinguished iRBD from HCs with a sensitivity of 91.9 ± 3.5% and a specificity of 90 ± 3.7%, however, the most salient feature was voice. Motor features contributed the following percentage of salient features: 26.67% from postural tremor, 10% from rest tremor, and 6.67% from gait and finger-tapping individually.
Barber et al, 2017, United Kingdom^18^	**Inclusion criteria**: for iRBD group: PSG-confirmed iRBD; individuals with concomitant OSA were only included if the two conditions were unequivocally distinguishable by PSG.	n = 171 (11.7% F); mean age = 64.7 ± 9.0; education = 13.7 ± 3.39; MMSE = 27.3 ± 2.10; MoCA = 25.1 ± 2.92	n = 296 (51% F); mean age = 64.9 ± 10.2; education = 15.1 ± 3.45; MMSE = 28.3 ± 1.89; MoCA = 26.7 ± 2.67	PD: n = 119 (29.4% F); mean age = 66.9 ± 9.1; education = 15.1 ± 3.86; MMSE = 27.6 ± 2.29 ; MoCA = 25.2 ± 3.32	UPDRS-III total score, Purdue Pegboard test score, Flamingo test score, TUG duration	N/A	Age and gender included as covariates; RBD group had a greater proportion of male participants. Additionally, Education, MMSE and MoCA were decreased in iRBD compared to controls.	Linear regression models; Logistic regression models	UPDRS-III score, Flamingo test score, and TUG duration were worse in iRBD compared to HCs. Purdue pegboard test performance was not different between groups.
Chen et al, 2014, China^20^	**Inclusion criteria**: for iRBD group: PSG-confirmed RBD (ICSD-II criteria) HCs: no history suggestive of RBD, Marburg RBD questionnaire score > 5 **Exclusion criteria**: Subjects with probable idiopathic PD or MSA, history of stroke or MSK impairment disorders, major depression, dementia, psychiatric diagnosis	n = 24 (29.2% F), mean age = 65.37 ± 8.50; disease duration = 9.13 ± 7.70; MMSE = 28.20 ± 1.79; MoCA = 25.83 ± 1.63	n = 23 (30.4% F); mean age = 64.21 ± 7.27; MMSE = 27.74 ± 1.66; MoCA = 26.74 ± 1.79	N/A	UPDRS-III total score Sway was assessed with a wearable inertial sensor (tri-axial accelerometers, 76 g, Opals, APDM Inc.) at the fourth and fifth lumbar vertebrae, while participants stood in upright position on a foot block placed on the floor for 30 s under 5 conditions: (i) feet together with eyes open condition, (ii) feet together with eyes closed, (iii) feet together, eyes open with dual task (subtracting by 3 s from 100), and (iv) feet together, eyes closed with dual task (subtracting by 3 s from 100), and (v) tandem standing with eyes open. JERK and RMS in AP, ML and on average measured.	N/A	None; Groups were not different in age, sex, and cognitive function (MMSE and MoCA).	ANOVAs	UPDRS-III scores were not different between groups. All sway parameters increased in iRBD compared to HCs in the eyes closed with dual task and tandem standing with eyes open conditions. All sway parameters except JERK and RMS in the ML direction were increased in iRBD compared to HCs in the eyes open with dual-task condition.
Cochen De Cock et al, 2020, France^21^	**Inclusion criteria**: for iRBD group: PSG-confirmed iRBD (ICSD-III criteria) **Exclusion criteria:** for iRBD group: drug-induced RBD, and presence of other neurological or neurodegenerative diseases for HCs: no history of neurological or psychiatric disorders, no sleep complaint, and no parkinsonian symptoms on physical examination	n = 21 (19% F); mean age = 68.7 ± 6.9; education = 12.5 ± 4.1; disease duration = 11.7 ± 11.85; MoCA = 26.2 ± 3.3	n = 38 (18.4% F); mean age = 69.1 ± 7.2; education = 12.9 ± 3.7; MoCA = 27.3 ± 1.9	PD: n = 38 (18.4% F); mean age = 69.1 ± 7.7; education = 13.4 ± 4.0; disease duration = 6.8 ± 4.5; MoCA = 26.4 ± 2.8	UPDRS-II and -III total scores, and scores for tremor and axial signs. Two tapping tests (taps recorded using digital drum pad (Roland HPD-20)): **Unpaced finger tapping test:** participants asked to tap for 60 s at their most comfortable rate with both hands in separate trials. Tapping rate (ITI) and variability (CV of ITI) measured. **Paced finger tapping test:** participants tapped with 1) sound of metronome and 2) to the beat of short musical excerpts. Variability (CV of ITI) measured.	N/A	None; participants were matched by age, sex, and education. MoCA was not different between groups.	T-tests	Participants with iRBD tapped faster than HCs in the unpaced tapping tests and tapped with more variability than HCs in both tests. Total scores on the UPDRS-II and -III did not differ, although iRBD exhibited significantly more tremor and axial signs.
Cochen De Cock et al, 2022, France^22^	**Inclusion criteria**: for iRBD group: PSG-confirmed RBD (ICSD-III criteria) **Exclusion criteria**: neurological or neurodegenerative diseases for iRBD group: drug-induced RBD	n = 21 (19% F); mean age = 68.7 ± 6.9; education = 12.5 ± 4.1; disease duration = 11.7 ± 11.85; MoCA = 26.2 ± 3.3	n = 21 (19% F); mean age = 69.8 ± 6.8; education = 12.8 ± 4.0; MoCA = 27.2 ± 2.1	PD: n = 21 (19% F); mean age = 69.8 ± 7.9; education = 13.5 ± 4.1; disease duration = 4.24 ± 2.77; MoCA = 26.6 ± 2.4	UPDRS-II and -III total scores. 20-m walking test conducted under single-task and dual-task (counting backwards from 100 by 1 s, and by 3 s) conditions while wearing 6 inertial measurement units (128 Hz; MobilityLab; APDM Inc., Portland, OR, USA) worn on both wrists, both ankles, sternal and lumbar positions. 59 gait parameters measured, representing stride length and velocity; cadence; gait cycle time; double support; swing and stance; ranges of motion; angular velocities of legs, arms, and trunk; and asymmetries of the stride, swing, stance, leg, and arm movements.	N/A	None; groups were matched by age, sex, and education; MoCA scores were not different between groups	Statistical machine learning methods (supervised classification models); Lasso linear regressions	UPDRS-II and -III scores were not different between groups. Gait outcomes distinguished iRBD from HCs with 95% accuracy (100% sensitivity and 91% specificity), with the greatest contribution from range of motion and asymmetry, peak swing velocity and asymmetry, range of motion, peak velocity, and phase difference and cadence.
Del Din et al, 2020, United Kingdom^23^	**Inclusion criteria**: for iRBD group: PSG-confirmed iRBD; individuals with concomitant OSA were only included if the two conditions were unequivocally distinguishable by PSG	n = 63 (7.9% F); mean age = 67.1 ± 9.4; disease duration = 4.5 ± 4.2; MMSE = 26.9 ± 2.2	n = 34 (0% F); mean age = 67.3 ± 10.1; MMSE = 28.6 ± 1.9	N/A	UPDRS-III total score, Purdue Pegboard Test score (duration), TUG duration, Falls rate in the past 6 months Free-living gait protocol: Participants wore tri-axial accelerometers (Axivity AX3) for one week on the fifth lumbar vertebrae. Ambulatory bouts (ABs, one bout = ≥4 steps) and step counts detected using a logical heuristics paradigm embedded into walking bout identification and quantification algorithm. Macro and Micro gait characteristics were determined. Macro behavior characteristics included: volume (total walking time per day, percentage of walking time per day, number of bouts and steps per day), pattern (mean bout length (generated based on AB detected over the 7 days), and non-linear descriptor (alpha, describes ABs distribution, ratio of short to long ABs (high alpha = greater ratio of short ABs to long ABs))), and variability (S2) of walking (within-subject variability of AB length; higher S2 means more varied walking activity pattern). Micro gait characteristics included: pace (step velocity (m/s), step length (m), swing time variability (s)), variability (step velocity variability (m/s), step length variability (m/s), step time variability (s), stance time variability (s)), rhythm (step time (s), swing time (s), stance time (s)), asymmetry (step time asymmetry (s), swing time asymmetry (s), stance time asymmetry (s)), postural control (step length asymmetry (m)).	N/A	Models adjusted for age, sex, MMSE, UPDRS-II and -III, and BMI	ANCOVAs; ROC analysis; logistic regression models	iRBD group had greater UPDRS-III scores compared to HCs and performed worse on the Purdue pegboard test and TUG. The iRBD group also had an increased falls rate compared to HCs. Mean bout length was significantly lower, and alpha was significantly greater in iRBD compared to HCs when looking at long bouts. Participants with iRBD walked slower with less variable velocity, and lower cadence: increased step time, swing time, and stance time, compared to HCs in total bouts, and in long bouts (with the exception of step velocity variability). These characteristics significantly discriminated RBD from HCs, particularly swing time.
Ehgoetz Martens et al, 2019, Australia^24^	**Inclusion criteria**: for iRBD group: history of dream enactment supported by nocturnal video PSG in accordance with SINBAR protocol	n = 24 (25% F); mean age = 66.9 ± 7.6; education = 14.6 ± 2.8; MoCA = 27.4 ± 2.2	n = 24 (42.8% F); mean age = 67.4 ± 10.1; education = 13.8 ± 2.8; MoCA = 28 ± 1.9	N/A	UPDRS-III scores Gait assessment: Participants walked on pressure sensor walkway (Zeno Walkway; Protokinetics, Havertown, PA; 6.1 m×0.61 m) under 5 different conditions: 1) normal pace, 2) fastest pace, 3) normal pace while counting backwards by 1 s, 4) normal pace while naming animals, 5) normal pace while counting backwards by 7 s. Velocity, step length, step time, CV of step length, step time asymmetry, step length asymmetry, step width, and step width CV were measured. Balance assessment: Participants stood on pressure sensor carpet (Zeno Walkway; Protokinetics, Havertown, PA) in a comfortable stance and performed 2 quiet stance trials for 30 s (eyes open and eyes closed), followed by 2 single-leg stance trials (on each leg). X and Y range, anterior-posterior and medial-lateral RMS of CoP displacement, mean CoP length, CoP velocity and maximum CoP velocity; total duration of single-leg stance trials were measured.	N/A	None; Groups did not differ in sex, age, education, or MoCA scores.	ANOVAs	UPDRS-III scores were greater in iRBD compared to HCs. All participants exhibited more impaired gait in the dual-task condition. Participants with iRBD exhibited increased step length asymmetry during fast-pace walking. HCs exhibited increased step width while dual-tasking, whereas participants with iRBD did not widen their step width, but they increased their step width variability significantly. There was a trend for participants with iRBD to exhibit increased anterior–posterior RMS during eyes-closed static balance, however, this did not remain significant after adjusting for multiple comparisons.

**Table:** 

Ehgoetz Martens et al, 2020, Australia^25^	**Inclusion criteria:** for iRBD group: PSG-confirmed RBD **Exclusion criteria:** No significant cognitive impairment (MMSE > 26), no other neurological disorders	n = 30 (20% F); mean age = 66.7 ± 7.2; education = 14.2 ± 2.7; MMSE = 29.1 ± 1.1; MoCA = 27.2 ± 2.2	n = 28 (50% F); mean age = 65.6 ± 8.1; education = 13.9 ± 3.2; MMSE = 29.2 ± 1.1; MoCA = 27.9 ± 1.8	N/A	1) VR gait paradigm performed while laying supine in an MRI machine, using foot pedals to navigate VR environment - under single-task and dual-task (cognitive cues presented as “STOP” or “WALK” as simple (STOP in red and WALK in green) or complex (Stroop)). Step time, step time variability, and stop sign response time measured. 2) overground walking on pressure sensor carpet (Zeno Walkway; Protokinetics, Havertown, PA) under single-task and dual-task (naming as many animals as possible) conditions. Step time and step time variability measured.	N/A	None; Groups were different in terms of sex, but not age, education, MMSE and MoCA.	ANOVAs	1) There was an interaction effect for stop sign response time, such that the HCs increased their step time in response to the complex cognitive task in VR, while participants with iRBD did not. 2) Step time variability increased under the dual-task condition (naming animals) but did not differ between groups. The iRBD group took longer steps during dual-task walking compared to HCs, but not during single-task walking
Ehgoetz Martens et al, 2022, Australia^26^	**Inclusion criteria:** for iRBD group: PSG-confirmed RBD **Exclusion criteria:** Satisfied criteria for PD (Berg et al, 2018), DLB (McKeith et al, 2017), MSA (Gilman et al, 2008)	n = 23 (82.6% F); mean age = 66.9 ± 7.2; education = 13.7 ± 2.5; MMSE = 29 ± 1.1; MoCA = 27.3 ± 2.2	n = 17 (52.9% F); mean age = 65.5 ± 8.2; education = 14 ± 3.4; MMSE = 29.3 ± 1.0; MoCA = 27.8 ± 1.6	Early PD: n = 16 (25% F); mean age = 62.3 ± 11.4; education = 13.7±;4.4 MMSE = 28.9 ± 1.5; MoCA = 28.1 ± 2.4	VR gait paradigm completed while laying supine in an MRI scanner with foot pedals placed at feet that allowed participants to navigate through VR environment. Walking and stopping in the virtual environment were initiated by simple and complex cue words that were briefly dis­played on the screen. Participants navigated through wide and narrow doorways in the VR environment. Modal footstep latency (i.e., step time) and variability (i.e., CV of step time) during doorway navigation (from 3 steps before and after the doorway) measured.	N/A	None; Groups were different in terms of sex, but not age, education, MMSE and MoCA.	ANOVAs	Participants with iRBD had longer step time overall. Both groups increased their step time in response to the narrow doorways compared to wide doorways, but this response was more exaggerated in iRBD compared to HCs. Both groups had greater step time variability while navigating narrow doorways, compared to wide doorways. Participants with iRBD had increased step time variability compared to HCs when navigating narrow doorways, but not wide doorways.
Geng et al, 2022, China^28^	**Inclusion criteria**: for iRBD group: v-PSG confirmed iRBD. **Exclusion criteria**: for iRBD group: cognitive impairment (MMSE < 26 scores); other neurological disorders, such as LBD and Parkinson's syndrome; patients with severe cardiac, pulmonary, hepatic, renal, and endocrine systemic diseases, and malignant tumors; history of psychiatric diseases, such as anxiety and depression; secondary RBD; other sleep disorders; contraindications to MRI examinations; taking medications including antipsychotics, electroconvulsive therapy (ECT), and alcohol-dependent patients; illiterate, deaf, etc. HCs: contraindications to MRI examinations; history of neuropsychiatric diseases and brain trauma; other sleep disorders; history of long-term alcohol abuse or other drug abuse	n = 21 (33.3% F), mean age = 61 ± 10.68; education = 8.81 ± 3.40; MMSE^a^ = 27 (26, 28)	n = 22 (40.9% F), mean age = 60.27 ± 7.60; education = 8.86 ± 3.14; MMSE^a^ = 28 (27, 28)	N/A	UPDRS-III score	N/A	Groups were matched by age and education; groups did not differ in terms of MMSE	T-tests	UPDRS-III scores were greater in the iRBD group than in HCs at baseline.
Iranzo et al, 2017, Spain^30^	**Inclusion criteria:** for iRBD group: PSG-confirmed RBD (excessive EMG activity in REM sleep associated with vigorous behaviors); no temporal association between introduction of medication and RBD symptoms onset **Exclusion criteria**: motor and cognitive problems, neurological diseases	n = 20 (20% F); mean age = 72.9 ± 8.6; education = 10.2 ± 4.6; disease duration = 12.1 ± 2.6	n = 32 (28.1% F); mean age = 69.5 ± 6.0; education = 10.1 ± 4.5	N/A	UPDRS-III total score	Mean follow-up: 12.1 ± 2.6 years	None; groups were matched by age and sex	T-tests	UPDRS-III scores were greater in the iRBD group than in HCs at baseline.
Kim et al, 2023, Republic of Korea^35^	**Inclusion criteria:** for iRBD group: PSG-confirmed RBD diagnosis (ASM criteria, n = 17) OR history of dream-enacting behavior (n = 4) for HCs: no RBD history or symptoms, normal results on neurological examination **Exclusion criteria**: neurodegenerative diseases, neurological disease due to secondary causes (stroke, seizure, brain tumor, psychiatric disorders, drug/toxic material-induced cognitive dysfunction, CNS infection, metabolic and endocrinological disorders, and hydrocephalus, etc.), and dementia	n = 21 (47.4% F); mean age = 68.7 ± 7.56; education = 9.6 ± 3.8; disease duration = 84.1 ± 41.0 months; MMSE score = 27.2 ± 3.1	n = 17 (52.6% F); mean age = 64.2 ± 6.7; education = 10.7 ± 4.0; MMSE score = 29.2 ± 1.4	N/A	UPDRS-III score, Finger-tapping capacity (from finger-tapping test instructions on the UPDRS), duration and number of steps during 5-m walk test.	N/A	None; groups did not differ in age or sex, but MoCA scores were significantly lower in iRBD compared to HCs	T-tests	There were no group differences in finger-tapping capacity, however, the iRBD group had greater UPDRS-III scores, and took longer to complete the walking test, with less steps.
Krupiçka et al, 2020, Czech Republic^36^	**Inclusion criteria**: for iRBD group: PSG confirmed RBD (ICSD-III criteria) > 49 years old for HCs: free of major neurologic disorders, active oncologic illness, and abuse of psychoactive substances. **Exclusion criteria**: for iRBD group: overt parkinsonism, dementia, and factors indicative of secondary RBD such as narcolepsy, drug-induced RBD, or focal brainstem lesions on MRI	n = 40 (12% F); mean age = 68 ± 6; disease duration = 5.4 ± 4.1; MoCA = 24.6 ± 4.8	n = 25 (12% F); mean age = 66 ± 7; MoCA = 25.8 ± 1.5	PD: n = 25 (20% F); mean age = 65.0 ± 8.0; disease duration = 1.4 ± 1.0: MoCA = 25.0 ± 2.8	UPDRS-III score Finger tapping test (according to UPDRS instructions) captured by contactless 3D motion capture system (V120, Trio: Optitrack) to track mutual distance of markers placed on distal phalanx of thumb and forefinger. Analyzed with BradykAn software. First 10 taps retained for subsequent analysis (in accordance with UPDRS instructions). Outcomes included amplitude decrement, mean opening velocity, and finger-tapping decrement (which represents the combination of the first two outcomes).	N/A	None; age, sex and MoCA scores did not differ between groups	ANOVAs; ROC analysis	UPDRS-III scores were greater in the iRBD group than in HCs, but the finger tapping item subscore did not differ. Despite this, iRBD had greater AD and FTD than HCs. AD and FTD had good diagnostic accuracy.
Lo et al, 2022, United Kingdom^37^	**Inclusion criteria:** > 18 years old, fluent in English, absence of cognitive impairment/dementia that would preclude the provision of consent for iRBD group: PSG-confirmed RBD (ICSD-III criteria)	n = 272 (12% F); mean age = 65.2 ± 8.8; disease duration = 1.3 ± 1.7; MoCA = 25.1 ± 2.8	n = 316 (47% F); mean age = 64.8 ± 10.1; MoCA = 26.7 ± 2.5	PD: n = 909 (36% F); mean age = 67.1 ± 9.6; disease duration = 1.2 ± 0.9; MoCA = 24.9 ± 3.3	UPDRS-III total score, TUG duration, Purdue pegboard test score (number of pins). A composite score was also calculated with the following formula: Composite clinical motor score = ((MDS-UPDRS-III-23.8829) × 0.0377 + (−1×Purdue + 28.9286) × 0.0739 + (TUG–9.8746) × 0.1414 + 3.8239) × 6.0946	Median follow-up: 3.5 years (only iRBD)	None	ROC analysis	The UPDRS-III had good discrimination accuracy and the highest discriminatory power for distinguishing iRBD and HCs. This was followed by the composite score, and then the TUG and Purdue pegboard test, although they all had relatively poor accuracy (AUC < 0.7).
Ma et al, 2021, China^38^	**Inclusion criteria:** for iRBD group: PSG-confirmed RBD; not diagnosed with any other neurodegenerative disease **Exclusion criteria:** for iRBD group: Total score <18 for RBDQ-HK, OSA syndrome or any other sleep disorder, musculoskeletal conditions, prior surgeries that could influence gait	n = 31 (16.1% F); mean age^a^ = 69 (63, 73); MoCA = 24 ± 3	n = 20 (55% F); mean age^a^ = 70 (67, 73); MoCA = 25 ± 3	N/A	UPDRS-III score Gait analysis with six wearable gyroscope and accelerometer sensors ((APDM; Mobility Lab, Portland, OR, United States) worn on wrists, ankles, anterior sternum, and lower back. Participants walked in corridor (10 m) at usual pace, fastest pace, and while doing a dual task (counting backwards from 100 by 7 s) for 1 min each. Gait outcomes included: normalized stride length (m), normalized stride velocity (m/s), stride time (s), range of motion of the trunk in sagittal plane (˚), range of motion of trunk in horizontal plane (˚), peak angular velocity of trunk in the sagittal or horizontal plane (˚/s), step time before turning (s), stride length asymmetry (m), CV of stride length (m), CV of stride time (s). Normalized values are normalized to participant height.	N/A	Analysis with normalized values adjusted for age and sex; Analysis for all other values adjusted for age, sex, and height.	ANCOVAs	UPDRS-III scores were greater in the iRBD group than in HCs. Participants with iRBD exhibited decreased trunk motion (reduced peak angular velocity and range of motion) while walking and increased step time before turning compared to HCs.
McDade et al, 2013, United States of America^39^	**Inclusion criteria:** Able to follow gait protocol, have informant who slept in the same room (MSQ) for pRBD group: satisfied MSQ criteria for pRBD **Exclusion criteria:** No dementia (DSM-IV criteria); stroke; subdural hemorrhage; history of alcohol abuse; PD; probable OSA	n = 42 (19% F); mean age^a^ = 79.0 (75.3, 84.1); MMSE^a^ = 28 (27,28); Visuospatial function z-score^a^ = 0.00 (20.66, 0.74); Memory function z-score^a^ = 0.32 (20.7, 1.2); Attention-executive function z-score^a^ = 0.24 (20.57, 0.81); Language function z-score^a^ = 0.05 (20.62, 0.58)	n = 492 (30% F); mean age^a^ = 79.4 (75.9, 84.0); MMSE^a^ = 28 (27, 29); Visuospatial function z-score^a^ = 0.44 (−0.19,0.94); Memory function z-score^a^ = 0.43 (−0.3, 1.2); Attention-executive function z-score^a^ = 0.4 (−0.12,0.97); Language function z-score^a^ = 0.4 (−0.12,0.97)	N/A	Gait analysis was performed using a pressure sensor walkway (GAITRite, 5.0 m×0.7 m, Sparta, NJ, USA). Participants walked at a normal pace with 1 m start-up distance, walking “up and back” for a total of 10 m. Gait outcomes included: cadence (steps/min), swing time (1/s), stance time (1/s), velocity (cm/s), double support time (1/s), stride length (cm), stride length SD (cm), stride time SD (s), stance time SD (s), swing time SD (s), double support time SD (s).	N/A	Model 1 adjusted for age and sex. Model 2 also adjusted for attention-executive function z-score. Model 3 also adjusted for Beck Depression inventory score, and visuospatial function z-score	Multivariate linear regression analyses	pRBD diagnosis was associated with reduced cadence, velocity, stride length, and increased stride time variability, swing time variability, and double support time. Velocity, stride length and stride time variability differences did not remain significant after adjusting for attention-executive function, BDI and visuospatial function scores.
Nepozitek et al, 2021, Czech Republic^40^	**Inclusion criteria:** for iRBD group: PSG confirmed RBD (ICSD-III criteria) for HCs: aged 50 or more years; no medical history of sleep or neurological disorders **Exclusion criteria:** for iRBD group: age under 50 years; clinical signs of overt dementia or parkinsonism; RBD associated with narcolepsy, encephalitis and head injury; presence of focal brain lesions found on MRI indicative of secondary RBD; use of anxiolytics or antidepressants following beginning of RBD symptoms	n = 34 (14.7% F); mean age = 67.7 ± 7.2; disease duration = 8.0 ± 6.6; MoCA = 23.4 ± 3.4	n = 33 (30.3% F); mean age = 61.5 ± 8.2; MoCA = 25.7 ± 2.3	N/A	UPDRS-II and UPDRS-III total scores	Mean follow-up for iRBD: 2.4 ± 1.5 years	None; age did not differ between groups; but MoCA was significantly greater in HCs than iRBD	T-tests and rank tests	UPDRS-II and UPDRS-III scores were greater at baseline in iRBD compared to HCs.
Nisser et al, 2022, Germany^41^	**Inclusion criteria**: for iRBD group: PSG-confirmed RBD (ICSD-II criteria) **Exclusion criteria**: Presence of parkinsonian motor signs (UK Brain Bank criteria), dementia (DSM-IV criteria), and other neurodegenerative diseases and comorbidities with known effects on motor function, such as stroke, orthopedic, rheumatoid, and pulmonary diseases, insulin-dependent diabetes mellitus and heart failure (New York Heart Association III-IV), and renal failure requiring dialysis.	n = 19 (31.6% F); mean age^b^ = 70.6 [68.7, 72.5], disease duration ^b^ = 7.1 [4.5, 9.7], MoCA^b^ = 27.5 [25.9, 29]	n = 20 (70% F); mean age^b^ = 64.4 [60, 68.7], MoCA^b^ = 28.6 [27.8, 29.4]	N/A	TUG duration, ATT score (number of taps), Grooved Pegboard test score (time to completion). Falling stick test: Catch a sudden plunging ruler stick with each hand. Average distance between initial position of stick and the position after it is caught with each hand measured. Bend, twist, and touch test: Standing with back to the wall, bend forward to touch a mark on the floor then back up to touch a mark on the wall behind. Total number of touches on the floor and wall in 20 s measured. 15- and 20- meter straight line walk test: Walk on a marked line forwards and backwards. Number of missteps measured. Force plate stance paradigm: Stand on force plate in bipedal stance, right pedal stance, and left pedal stance. Total CoP excursion measured.	N/A	Adjusted for age; sex and MMSE did not differ between groups	ANCOVAs/ MANCOVAs; ROC analysis; multivariate backward logistic regression modeling	The iRBD group exhibited worse performance in the falling stick test, backwards straight line walk tests (15 and 20 m) than the HCs. They also performed worse on the bend, twist, and touch test, however, this did not remain significant after correction for multiple comparisons. The TUG and ATT showed poor discrimination accuracy. The falling stick test demonstrated the best discrimination accuracy. The bend, twist and touch test, grooved pegboard test, and backwards straight-line test showed good diagnostic performance.
Pereira et al, 2019, Sweden^42^	**Inclusion criteria:** for iRBD group: PSG-confirmed, free of neurological diseases for HCs: no significant neurological dysfunctions, first-degree family members with PD, or cognitive impairment (MoCA; score ≥27) PD: standard diagnostic criteria for PD, diagnosed within 2 years, untreated for PD, and significant DaTscan deficit **Exclusion criteria:** for HCs: ≥ 5 RBDSQ score, significant olfactory dysfunction for their age and sex	n = 27 (18.5% F); mean age = 68.9 ± 5.5; education = 12.7 ± 5.2; MoCA = 25.3 ± 4.5	n = 31 (35.5% F); mean age = 58.5 ± 11.0; education = 16.5 ± 3.1; MoCA = 28.3 ± 1.2	PD: n = 151 (37.7% F); mean age = 60.6 ± 9.6; education = 15.4 ± 2.9; MoCA = 27.3 ± 2.3	UPDRS-III total score	Converters, mean follow-up: 2.9 ± 0.5 years Non-converters, mean follow-up; 2.8 ± 0.5	Adjusted for age and sex; MoCA scores and years of education did not differ between groups	ANOVAs	UPDRS-III scores were greater in iRBD than HCs at baseline.
Postuma et al, 2006, Canada^43^	**Inclusion criteria**: RBD group: PSG-confirmed RBD (ICSD-II) **Exclusion criteria**: probable idiopathic PD (UK brain bank criteria) or MSA, or dementia (MMSE < 24)	n = 25 (12% F); mean age^c^ = 69.2 (44–93); disease duration^c^ = 10.5 (2–30); MMSE^c^ = 28.4 (27–30)	n = 25 (12% F); mean age^c^ = 69.2 (46–87); MMSE^c^ = 28.8 (27–30)	N/A	UPDRS-III total score, TUG duration, ATT score (number of taps), Purdue pegboard test score (number of pins)	N/A	None; Groups were matched by age and sex.	T-tests	The iRBD group had greater UPDRS-III scores, and performed worse on the ATT and TUG than HCs. There was no difference in the Purdue pegboard performance.
Postuma et al, 2009, Canada^44^	**Inclusion criteria**: for iRBD group: PSG-confirmed RBD (ICSD-II criteria) for HCs: PSG confirming the absence of RBD **Exclusion criteria**: Dementia (significant impairment on at least two cognitive domains (executive functions and attention, verbal learning and memory, or visuo-spatial abilities) on neuropsychological testing in association with functional impairment due to cognitive impairment).	n = 68 (22% F); mean age^c^ = 68.0 (44–93); MMSE = 28.0 ± 0.26	n = 36 (22.2% F); mean age^c^ = 65.8 (46–87); MMSE = 29.2 ± 0.30	PD with RBD: n = 34 (18% F); mean age^c^ = 68.8(49–94); MMSE = 28.4 ± .24 PD without RBD: n = 21 (38% F); mean age^c^ = 69.8(49–83); MMSE = 29.1 ± 0.22	UPDRS-II and III total scores, TUG duration, ATT score (number of taps), Purdue pegboard test score (number of pins)	N/A	None; Groups were matched by age and sex.	ANOVAs	UPDRS-II scores were greater in iRBD compared to HCs, but not UPDRS-III. Participants with iRBD performed worse on the TUG, ATT, and the Purdue pegboard test.
Simonet et al, 2024, United Kingdom^46^	**Inclusion criteria**: for iRBD group: PSG confirmed RBD **Exclusion criteria**: Formal diagnosis of dementia, PD, essential tremor, motor neuron disease, MS, polyneuropathy	n = 33 (9.1% F); mean age = 68.66 ± 8.07; disease duration = 10.6 ± 6.87	n = 29 (13.9% F); mean age = 69.65 ± 7.74	N/A	UPDRS-III total score Duration of a 10 m walking test under single and dual task (listing months of the year in reverse order, subtracting from 100 by 3 s) conditions BRAIN: alternate tapping of the ‘s’ and ‘;’ keys with the index finger. DFT: repeated tapping of down arrow key with left index finger whilst depressing the left arrow key with left middle finger. Kinesia score (number of keystrokes), akinesia time (average dwell time keys are depressed), and incoordination score (variance of traveling time between keystrokes) were measured for BRAIN and DFT. FT-SMART: Relative amplitude and velocity of finger-tapping under task single-task and dual task (listing months of the year in reverse order, subtracting from 100 by 3 s) conditions.	N/A	None; age and sex did not differ between groups	Welch's test; logistic regression models and ROC analysis; multivariate logistic modeling	UPDRS-III scores were greater in the iRBD group than in HCs. The iRBD group took longer to complete the 10-m walking test while dual-tasking compared to HCs. Participants with iRBD had reduced kinesia scores, and greater akinesia and incoordination scores than HCs in the BRAIN and DFT tests. There were no differences in the FT-SMART task during the single-task condition, however, iRBD exhibited slower, more erratic tapping with lower amplitude while dual-tasking compared to HCs.
Viteckova et al, 2020, Czech Republic^48^	**Inclusion criteria**: for iRBD group: PSG-confirmed RBD (ICSD-III criteria) **Exclusion criteria**: History of neurological disorders	n = 67 (13.4% F); mean age = 66.22 ± 8.39; education = 14.4 ± 3.3; MoCA = 26.3 ± 3.0	n = 40 (15% F); mean age = 64.23 ± 8.23; education = 15.1 ± 3.4; MoCA = 25.3 ± 2.3	N/A	Participants performed the TUG on a pressure sensor walkway (GaitRite) under single task, motor dual task (carrying glass of water), and cognitive dual task (serial 3 s, with no task prioritization) conditions. Outcomes included: velocity, step length, step time, step width, gait cycle time, swing (%), stance (%), single support (%), double support (%), CV of step length, CV of step width, step length asymmetry, step time asymmetry	N/A	None	T-tests	There were no significant group differences between groups on any of the gait variables in any of the conditions.
Wan et al, 2016, China^49^	**Inclusion criteria:** for iRBD group: PSG-confirmed RBD (ICSD-II criteria); score on the RBDQ-HK > 17 **Exclusion criteria**: dementia (defined as MMSE <24 with functional impairment); a past history of psychiatric illness and treatment with antidepressants; a past history of other sleep disorders including OSA syndrome, periodic limb movement during sleep, and narcolepsy; a past history of nasal cavity operation, chronic rhinitis, use of other medicinal remedies, abuse of alcohol and tobacco	n = 41 (41.5% F); mean age = 67 ± 8.9; disease duration = 9.5 ± 7; MMSE^a^ = 29 (27, 29.5)	n = 63 (47.6% F); mean age = 67.9 ± 7; MMSE^a^ = 29 (28, 30)	PD with RBD: n = 42 (35.7%); mean age = 69.7 ± 6.4; MMSE^a^ = 29 (27.8, 30) PD without RBD: n = 35 (37.1%F); mean age = 67.8 ± 8.1; MMSE^a^ = 29 (27, 29)	UPDRS-III total score, TUG duration, ATT score (number of taps), Purdue pegboard test score (number of pins)	N/A	None; Age, gender, MMSE were not different between groups	ANOVAs	The iRBD group had greater UPDRS-III score, and performed worse on the ATT, Purdue pegboard test, and the TUG than HCs.

**Table:** 

Zatti et al, 2024, Italy^51^	**Inclusion criteria**: for iRBD group: history of dream-enacting behavior; PSG proven REM sleep with sustained electromyographic activity; absence of known pathological neurologic condition; lack of motor or cognitive complaints, normal MRI, and lack of another sleep disorder, medical disorder or medication interfering with gait, or substance abuse; lack of abnormalities of gait at standard neurological examination.	n = 23 (17% F); mean age = 72 ± 6; disease duration = 4 ± 3; MoCA = 25 ± 3	n = 65 (60% F); mean age = 69 ± 6; MoCA = 27 ± 2	PD: n = 60 (47% F); mean age = 68 ± 8; disease duration = 1 ± 1; MoCA = 24 ± 3	TUG performed at normal and fast walking speed with Rehagait inertial movement unit placed on 5th lumbar vertebrae. TUG duration, duration of turns, mean angular velocity, and peak angular velocity under normal speed and fast speed were measured, as well as amount (%) of variation of TUG duration, turning duration, mean velocity, and peak velocity between conditions.	N/A	Adjusted for age and sex in analyses	ANCOVAs	Participants with iRBD exhibited longer turn duration and lower mean and peak angular velocities compared to HCs, but only during normal speed TUG. The iRBD group experienced a greater increase in TUG speed between normal and fast TUG than HCs.
Zhang et al, 2021, China^52^	**Inclusion criteria**: Right-handed, > 18 years old for iRBD group: PSG-confirmed RBD (ICSD-III criteria) **Exclusion criteria**: History of psychiatric disorders, stroke, head trauma, unstable hypertension or diabetes, chronic obstructive pulmonary disease, or electroencephalographic abnormalities suggesting epilepsy, encephalitis, or any other neurological conditions	n = 21 (28.5% F), mean age = 65.3 ± 7, education = 11.7 ± 4.5, disease duration = 5.7 ± 5	n = 28 (43.7% F), mean age = 62.9 ± 5.1, education = 11.2 ± 3.1	N/A	TUG duration, ATT (number of taps), Flamingo test (median score)	N/A	Groups were matched by age, sex, and education	T-tests; rank sum tests	The iRBD group had worse performance on ATT (average and both hands), and TUG compared to HCs. Performance on the flamingo test was not different between groups.
Zhang et al, 2023a, China^53^	**Inclusion criteria**: for iRBD group: PSG confirmed RBD (ICSD-III criteria) for HCs: no RBD, neurological diseases or mental disorders **Exclusion criteria**: Clinical diagnosis of PD, MSA, dementia, other forms of neurodegenerative disorders, secondary RBD due to medical condition, drug-induced RBD, RBD mimics, history of stroke, epilepsy, encephalitis, or any other neurologic disorders, psychiatric disorders, head trauma, unstable hypertension or diabetes, chronic obstructive pulmonary disease, video-PSG confirmed severe OSA with apnea-hypopnea index >30	Converters: n = 21 (38.1% F); mean age = 66.42 ± 5.71; education = 12 ± 8; disease duration = 4.54 ± 5.03; MMSE z-score: -1.43 ± 1.92 Non-converters: n = 24 (12.5% F); mean age = 64.50 ± 5.12; education = 12.5 ± 3; disease duration = 4.52 ± 3.07; MMSE z-score: 1.43 ± 2.56	n = 25 (24.0% F); mean age = 64 ± 3.79; education: 11.5 ± 3; MMSE z-score: -0.27 ± 1.92	N/A	TUG duration, ATT score. Both were transformed to z-scores based on mean and SD of HCs.	Converters: 2 ± 2.3 years Non-converters: 4 ± 2.4 years	Participants matched by age and sex; Groups did not differ on MMSE score	T-tests; ANOVAs; ROC analysis	ATT performance did not differ between participants with iRBD and HCs, however, both iRBD groups performed worse on the TUG than HCs.
Zhang et al, 2023b, China^54^	**Inclusion criteria:** for iRBD group: v-PSG confirmed iRBD (ICSD-III criteria) PD + RBD: MDS PD criteria, typical self-reported dream enactment behavior before the diagnosis of PD **Exclusion criteria:** age more than 79 years; history of stroke, epilepsy, encephalitis, or any other neurologic disorders, psychiatric disorders, head trauma, unstable hypertension or diabetes, chronic obstructive pulmonary disease	n = 32 (32.53% F); mean age = 68.79 ± 5.17; disease duration (time since RBD onset) = 9.97 ± 6.41; MoCA^a^ = 27.00 (24.00, 28.00)	n = 83 (15.62% F); mean age = 68.92 ± 5.09; MoCA^a^ = 28.00 (27.00, 29.00)	PD with RBD: n = 80 (22.50% F); mean age = 67.55 ± 5.86; MoCA^a^ = 27.00 (25.00, 28.00)	Total UPDRS-III score	N/A	None	T-tests	UPDRS-III scores were greater in iRBD than HCs at baseline.
**Longitudinal**									
Campabadal et al, 2020, Spain^19^	**Inclusion criteria:** for iRBD group: history of dream reenactment behavior, v-PSG demonstration of RSWA, absence of motor complaints at time of recruitment, unremarkable neurological exam, normal brain MRI, no temporal association between estimated RBD onset and introduction or withdrawal of medications for HCs: no sleep disorders, no cognitive or motor impairment **Exclusion criteria:** clinical evidence of movement disorder, presence of psychiatric and/or neurologic comorbidity; low global IQ score (determined as scalar score ≤ 7points on the Vocabulary subtest of the Wechsler Adult Intelligence Scale, 3rd edition); global cognitive impairment (determined as MMSE score < 25), claustrophobia, and MRI movement artifacts.	n = 14 (21.4% F); mean age = 70.1 ± 6.0; education = 10.1 ± 5.1; disease duration = 4.5 ± 3.4; age at iRBD onset = 65.6 ± 7.5; MMSE = 27.9 ± 1.7	n = 18 (61.1% F); mean age = 68.3 ± 7.5; education = 10.9 ± 4.2; MMSE = 29.4 ± 1.0	N/A	UPDRS-III score	iRBD: average follow-up = 1.6 ± 0.3 HCs: average follow-up = 1.6 ± 0.2	Models were adjusted for sex.	Linear mixed models	No significant effect of time was found for the UPDRS-III scores in the iRBD group.
Fereshtehnejad et al, 2019, Canada^27^	**Inclusion criteria:** for iRBD group: PSG-confirmed RBD **Exclusion criteria**: Parkinsonism and/or dementia	Provided only for individuals that phenoconverted: n = 55 (30.9% F); mean age = 64.6 ± 9.5; disease duration = 8.2 ± 9.0	-		UPDRS-II and III total score, TUG duration, ATT score, Purdue pegboard test score	9 years (for iRBD only)	Models adjusted for baseline age.	T-tests; regression modeling; linear mixed effects modeling; ROC analysis	Deficits in the ATT were first to deviate from normal, estimated to appear 12.9 years before phenoconversion, followed by UPDRS-II scores(9.3 years before phenoconversion), then Purdue pegboard test performance (7.5 years before conversion), and finally UPDRS-III scoresand TUG performance (6.5 years before phenoconversion). UPDRS-II and -II had the highest accuracy at distinguishing iRBD who had phenoconverted from HCs, followed by ATT, then Purdue pegboard test and TUG. Tests became less sensitive as the prodromal interval decreased. The ATT remained sensitive (>55%) up to 6 years before phenoconversion, while the UPDRS-II and -III did not remain sensitive.
Han et al, 2021, China^29^	**Inclusion criteria:** Participants in the Beijing Longitudinal Study on Aging II: aged 55 years or older, with 1-year follow-up data available for pRBD group: RBDQ-HK score > 19	n = 6891 (61.4% F), mean age = 71.4	N/A	N/A	Subjects were classified as fallers according to a numerical answer greater than zero to the question “During the preceding 12 months, how many times have you unintentionally lost your balance and land on the ground or lower level?” Risk of falls (OR) calculated.	1 year	**Model 1**: adjusted for age and sex; **Model 2**: adjusted for age, sex, education, marital status, occupation, residence type, family income, smoking, drinking, physical activity, protein intake, fruits and vegetables intake, BMI, family history of parkinsonism or dementia, fall history, and various clinical comorbidities (stroke, CHD, hypertension, diabetes, hyperlipidemia, hyperuricemia, visual impairment, hunchback, cognitive impairment, depression, ADL score, IADL score); **Model 3**: adjusted for all the above covariates and fear of falling and gait and balance impairment.	Logistic regression models	Presence of pRBD was associated with a 2.57-fold risk of falling than elderly without pRBD, after adjusting for age and sex. In model 2 and 3, the OR was reduced to 1.98 and 1.75, respectively, and remained significant.
Janzen et al, 2022a, Germany^31^	**Inclusion criteria:** v-PSG confirmed RBD **Exclusion criteria**: history of (other) neurological diseases, diabetes mellitus, hyperthyroidism or hypothyroidism, stroke, significant head trauma, or other relevant comorbidities	normal MIBG: n = 5 (20% F); mean age = 60.9 ± 6.4; disease duration^a^ = 10.0 (7.6, 19.2); MoCA^a^ = 27.0 (24.5, 29.0) pathological MIBG: n = 12 (8% F); mean age = 63.5 ± 5.3; disease duration^a^ = 7.8 (6.2, 9.6); MoCA^a^ = 27.0 (26.0, 28.0)	N/A	N/A	UPDRS-III total score	normal MIBG: mean follow-up = 3.7 years pathological MIBG = mean follow-up = 3.6 years	None	T-tests and rank tests	UPDRS-III did not change after approximately four years of follow-up in the normal MIBG or abnormal MIBG groups.
Janzen et al, 2022b, Germany^32^	**Inclusion criteria:** v-PSG confirmed iRBD **Exclusion criteria**: Subjects with diseases (heart/kidney failure, myocardial infarction within the last five years, diabetes, amyloid or other neuropathy, pheochromocytoma) and/or intake of certain medications (reserpine, opioids, labetalol, phenylpropanolamine, phenylephrine) which may affect [123I] MIBG	normal MIBG: n = 8 (25% F); mean age^a^ = 58.5 (53.8, 65.0); disease duration^a^ = 57.0 (12.5, 113.3) months; age at iRBD diagnosis^a^ = 58.5 (53.8, 65.0); MoCA^a^ = 26.5 (24.3, 28.5) pathological MIBG + normal FP-CIT: n = 9 (11.1% F); mean age^a^ = 66.0 (57.5, 71.0); disease duration^a^ = 55 (29.5, 151.0) months; age at iRBD diagnosis^a^ = 66.0 (57.5, 71.0); MoCA^a^ = 26.0 (24.0, 28.5) pathological MIBG + FP-CIT: n = 29 (10.3% F); mean age^a^ = 66.0 (62.5, 71.5); disease duration^a^ = 59.0 (25.5, 102.5) months; age at iRBD diagnosis^a^ = 59.0 (25.5, 102.5); MoCA^a^ = 27.0 (25.3, 28.8)	N/A	N/A	UPDRS-III total score	49.1 ± 18.6 months	None	T-tests and rank tests	When participants were divided into normal and pathological MIBG groups, UPDRS-III significantly increased after follow-up in only those with iRBD and pathological MIBG. When the pathological MIBG group was further subdivided based on whether FP-CIT was normal or abnormal, UPDRS-III significantly increased after follow-up in only those with iRBD and pathological MIBG and FP-CIT.
Joza et al, 2023, Canada^33^	**Inclusion criteria:** PSG-confirmed iRBD according to standard criteria; without parkinsonism or dementia at baseline; baseline assessment and at least one follow-up visit; subjects were required to meet MDS research criteria for probable prodromal PD, defined according to the criteria as having at least an 80% probability of prodromal PD	n = 1160 (21.6% F); mean age = 68.5 ± 7.0; time from iRBD diagnosis = 1.28 ± 2.3; self-reported iRBD duration = 6.4 ± 6.4	N/A	N/A	UPDRS-II and UPDRS-III total scores, TUG duration, Purdue Pegboard	Mean follow-up = 3.3 ± 2.2 years	Models adjusted for baseline age.	Linear mixed modeling	All motor assessments showed clear progression over time. The UPDRS-III showed the greatest degree of progression, followed by the Purdue Pegboard test, UPDRS-II and finally TUG duration.
Kim et al, 2022, South Korea^34^	**Inclusion criteria**: for iRBD group: v-PSG confirmed iRBD; free of dementia and parkinsonism; secondary cause of RBD was ruled out for PD + RBD group: newly diagnosed based on UK PD Brain Bank criteria without previous PD medication; had probable RBD diagnosis via RBD screening questionnaire score >6 **Exclusion criteria**: included white matter changes greater than grade I small vessel disease, space-occupying lesions, structural lesions revealed on conventional brain MRI; history of nasal or sinus diseases; history of depression or other psychiatric illness; presence of other neurological diseases; presence of dementia or symptoms of cognitive fluctuation and visual hallucination suggesting DLB; patients taking antipsychotics or antidepressants that affect the dopamine and serotonin systems	n = 28 (43% F); mean age = 69.8 ± 5.7; disease duration = 4.7 ± 3.9; age at iRBD diagnosis = 65.1 ± 7.1; MMSE = 27.5 ± 2.2	n = 28 (64% F); mean age = 70.2 ± 4.4; MMSE = 28.4 ± 1.5	PD + RBD: n = 24 (42% F); mean age = 69.6 ± 7.5; disease (PD) duration = 1.2 ± 1.4; RBD duration = 5.3 ± 6.2; MMSE = 27.0 ± 2.9	UPDRS-II and UPDRS-III total scores	Median follow-up for iRBD group^c^ = 5.1 (1.5, 8)	None	T-tests and rank tests	UPDRS-II and UPDRS-III scores increased after follow-up in the iRBD group.
Postuma et al, 2012, Canada^45^	**Inclusion criteria:** PSG-confirmed iRBD (ICSD-II criteria); free of parkinsonism and dementia at enrolment; complete baseline examination including quantitative motor markers **Exclusion criteria:** presence of dementia, defined as significant impairment on at least two cognitive domains (executive functions and attention, verbal learning and memory, or visuospatial abilities) on neuropsychological testing in association with functional impairment due to cognitive impairment	n = 20 (25% F); mean age = 70.5 ± 6.9; time from iRBD diagnosis = 2.8 ± 3.0; RBD symptom duration = 6.5 ± 4.0	N/A	N/A	UPDRS (1987 version) score, divided by cardinal manifestations (bradykinesia/akinesia, rigidity, rest tremor, and gait disorder, ATT, Purdue Pegboard, TUG duration	Mean follow-up: 3.3 ± 1.8 years	None	Regression analysis, ROC analysis	UPDRS (1987) scores deviated from normal values approximately 4.5 years before phenoconversion. Specifically voice and face akinesia were the first to deviate, followed by rigidity, gait, limb bradykinesia and tremor. The Purdue pegboard test and ATT deviated from normal approximately 8 years before phenoconversion, and TUG duration deviated approximately 6.3 years from phenoconversion. ATT showed the greatest degree of progression over time, followed by total UPDRS. The results of the ROC analysis showed that the ATT was the most accurate and sensitive outcome for prediction of phenoconversion at 3 years before phenoconversion. On the other hand, TUG was the most specific outcome at 3 years before phenoconversion. UPDRS score was more accurate and specific than ATT at 1 and 2 years before phenoconversion, but not at 3 years.
Stær et al, 2023, Denmark^47^	**Inclusion criteria:** PSG-confirmed iRBD **Exclusion criteria:** Presence of MCI, dementia, parkinsonism, and other neurological disorders for HCs: presence for iRBD symptoms	n = 12 (20% F); mean age = 64.9 ± 5.5; disease duration = 3.5 ± 2.8; MMSE = 28.3 ± 1.7; MoCA = 25.3 ± 2.3	n = 9 (0% F); mean age = 64.3 ± 6.9; MMSE = 29.6 ± 0.7; MoCA = 26.8 ± 2.7	N/A	UPDRS-III total score	Mean follow-up for iRBD group: 3.08 ± 0.46 years	None	Paired t-tests	UPDRS-III scores increased from baseline to follow-up in the participants with iRBD.
Woo et al, 2024; South Korea^50^	**Inclusion criteria:** v-PSG confirmed iRBD; no dementia or parkinsonism **Exclusion criteria:** Subjects with depression or psychiatric disorders, structural or vascular brain damage, and severe obstructive sleep apnea	iRBD with constipation: n = 29 (41.4% F); mean age = 72.10 ± 6.26; education = 7.64 ± 4.49; MMSE = 26.10 ± 3.22 iRBD without constipation: n = 24 (54.2% F); mean age = 67.58 ± 7.77; education = 10.42 ± 4.90; MMSE = 26.67 ± 3.63	N/A	N/A	UPDRS-II and UPDRS-III total scores	Mean follow-up for all iRBD: 4.08 ± 2.64	Models adjusted for baseline age and sex.	linear generalized-estimating-equations regression analysis	UPDRS-II and UPDRS-III scores increased over time significantly in all iRBD groups. The yearly progression of these motor outcomes worsened over time at a greater rate in those with iRBD with constipation, than those with iRBD without constipation.

Abbreviations: ADL: Activities of daily living; ANOVA: analysis of variance; ANCOVA: analysis of covariance; ATT: Alternate tap test; BRAIN: Bradykinesia Akinesia Incoordination; BMI: Body mass index; CHD: Congenital heart disease; CoP: Centre of Pressure; CV: Coefficient of Variation; DFT: Distal finger tapping; EMG: Electromyography; FP-CIT: Fluoropropyl-Carbomethoxy-Iodophenyl-Tropane; FOG: Freezing of gait; FT-SMART: Finger Tapping - Slow Motion Analysis of Repetitive Tapping; GRF: Ground reaction forces; HCs: HCs; IADL: Instrumental activities of daily living; iRBD: isolated REM sleep behavior disorder; ITI: inter-tap interval; MIBG: meta-iodobenzylguanidine; MMSE: Mini-Mental State Examination; MoCA: Montreal Cognitive Assessment; MRI: Magnetic resonance imaging; MSQ: Mayo sleep questionnaire; OSA: Obstructive sleep apnea/hypopnea syndrome; PD: Parkinson's Disease; pRBD: Probable REM Sleep Behavior Disorder; PSG: polysomnography; RBDQ-HK: REM sleep behavior disorder questionnaire-Hong Kong, RMS: root mean square; ROC: receiver operating characteristics; RSWA: REM Sleep without Atonia; SD: standard deviation; TA: tibialis anterior; TUG: Timed Up and Go; UPDRS: Unified Parkinson's Disease Rating Scale, VR: virtual-reality.

Age, education, and disease duration are in years, unless stated otherwise.

Participant characteristics data presented as mean ± standard deviation unless stated otherwise below:

^a^ Data is presented as median (upper quartile, lower quartile)

^b^ Data is represented as mean [95% CI]

^c^ Data is presented as median (range)

#### UPDRS

Four studies assessed motor complications in daily living using the UPDRS-II.^21,22,40,44^ Of these studies, two studies found that UPDRS-II scores were greater in iRBD compared to HCs,^40,44^ and two did not find any group differences.^21,22^

The UPDRS-III was assessed by 20 studies.^16,18,20–24,28,30,35–38,40,42–44,46,49,54^ Of these studies, 14 reported that UPDRS-III scores were greater in iRBD compared to HCs,^18,23,24,28,30,35,36,38,40,42,43,46,49,54^ and five reported that UPDRS-III scores were not different between participants with iRBD and HCs.^16,20–22,44^ It is worth noting that in the studies that did not detect differences, motor abnormalities were detected through other motor assessments.^16,20–22,44^ Results of these studies are discussed in greater detail in later sections.

Some studies also performed discriminatory analyses to investigate the diagnostic accuracy of the UPDRS-II and III at discriminating iRBD from HCs, or iRBD that phenoconverted from HCs.^36,37,46^ One study reported that the UPDRS-III had good discrimination accuracy for iRBD from HCs.^37^ Other studies showed the UPDRS-III has good accuracy at discriminating iRBD from HCs, however, it was less accurate than other quantitative motor assessments.^36,46^

Details of all studies that assessed UPDRS-II and UPDRS-III are summarized in Supplemental Table 1.

#### Tremor

Tremor was assessed and compared in three studies, which are summarized in Supplemental Table 2.^17,21,22^ One study used a smartphone with inertial measurement units to measure postural and rest tremor, which was found to be the most salient features that could discriminate iRBD from HCs.^17^ Two other studies also found tremor, measured by the UPDRS-III, to be greater in iRBD than HCs.^21,22^

#### Tapping assessments

Several different tapping assessments were performed, namely the alternate tap test, finger-tapping assessments, and keyboard tapping tests, in a total of 11 studies.^17,21,35,36,41,43,44,46,49,52,53^ We subdivided tapping assessments into clinical assessments of tapping (e.g., UPDRS-III finger-tapping item, alternate tap test) and quantitative assessments (e.g., motion capture analysis). These studies are summarized in Supplemental Table 3.

Two studies assessed finger-tapping using the UPDRS-III finger-tapping item, with both finding that scores did not differ between groups.^35,36^ Additionally, six studies used the alternate tap test,^41,43,44,49,52,53^ four of which reported that iRBD performed worse than HCs,^43,44,49,52^ and two did not.^41,53^

Four studies assessed tapping quantitatively.^17,21,36,46^ One study used an alternate finger-tapping test on a smartphone, finding that finger-tapping task outcomes were among the most salient features that discriminated iRBD from HCs.^17^ Another study found participants demonstrated more arrhythmic tapping during unpaced and paced tapping, as well as faster tapping in the unpaced test.^21^ Another study used the UPDRS-III finger-tapping test instructions but assessed finger-tapping with a motion capture system, reporting that participants with iRBD exhibited amplitude decrements in their finger-tapping compared to HCs, which were not captured in the UPDRS-III finger-tapping item subscore.^36^ It is also worth noting that the finger-tapping outcomes from the motion capture analysis exhibited better accuracy than the UPDRS-III.^36^

Finally, another study also used motion capture to assess finger-tapping using the Slow-Motion Analysis of Repetitive Tapping (SMART), and two keyboard tests, namely the BRadykinesia Akinesia INcoordination test (BRAIN) and Distal Finger Tapping test (DFT). SMART refers to a video-based tool which tracks repetitive finger tapping that follows the UPDRS-III instructions.^46^ This was done alone and with a secondary task of counting backwards by 3s. BRAIN requires participants to alternate between tapping the “s” and “;” keys on a keyboard with one index finger for 30 seconds, whereas DFT requires participants to repeatedly tap the down arrow key with their left index finger while depressing the left arrow key with their left middle finger for 20 seconds. Worse performance was found in iRBD compared to HCs under dual-tasking conditions, specifically participants with iRBD exhibited slower and more erratic tapping with lower amplitude than controls.^46^ This study also found that participants with iRBD performed worse on the keyboard tapping tests as they had lower kinesia (less keystrokes), greater akinesia (greater dwell time when keys were depressed), and more incoordination in their keyboard tapping.^46^

#### Reaction time

Reaction time was assessed and compared in two studies, which are summarized in Supplemental Table 4.^17,41^ One study used a smartphone application and found that reaction time outcomes were not among the most salient features that could discriminate iRBD from HCs.^17^ On the other hand, another study evaluated reaction time using the falling stick test, and found it was significantly different between groups and demonstrated strong discrimination accuracy.^41^ The falling stick test involves the participant catching a plunging stick as it was dropped by the examiner without any notice. The difference between the initial position of the stick and the position after griping the stick is measured and averaged across three trials for each hand.

#### Purdue pegboard test

The Purdue pegboard test was assessed and compared by seven studies, one of which used the Grooved pegboard test.^18,23,37,41,43,44,49^ These studies are summarized in Supplemental Table 5. Three studies found that participants with iRBD performed worse than HCs,^23,44,49^ three found that performance did not differ,^18,41,43^ and one reported the Purdue Pegboard test had poor discrimination accuracy between iRBD and HCs.^37^

#### Balance assessments

Balance was assessed by eight studies total, and assessments included the Flamingo test,^18,23,52^ quantitative analyses of balance on a smartphone,^17^ force plates,^41^ a pressure sensor carpet,^24^ and with wearable sensors.^20^ One study used a dynamic balance test.^41^ All studies are summarized in Supplemental Table 6.

Three studies used the Flamingo test, with only one finding that the iRBD group performed worse than HCs,^18^ while the other two studies did not report a difference.^23,52^ The flamingo test evaluates the participant's ability to stand on one leg for a given amount of time. In two of these studies, participants were asked to stand on one leg for 30 seconds, while in the other study, participants were asked to stand on one leg for one minute.

Quantitative balance assessments were conducted by four studies.^17,20,24,41^ One study used a smartphone application and found that balance outcomes were among the most salient features that could distinguish iRBD from HCs.^17^ One study used wearable sensors to measure postural sway parameters, finding that these measures were worse in iRBD compared to HCs under dual-tasking conditions (subtracting from 100 by 3s) with eyes open and eyes closed, as well as during tandem standing.^20^ Another study assessed balance on a pressure sensor carpet and found only marginal differences between groups in root mean square (RMS) when standing with eyes closed.^24^ Finally, a study used force plates and measured total excursion of center of pressure when standing in bipedal or unipedal stances, but no differences were identified between groups.^41^

One study used a dynamic balance test where participants walked forwards and backwards on a straight line marked on the floor and the number of missteps were recorded.^41^ Researchers found worse dynamic balance in iRBD when walking backwards, and that this test had good discrimination accuracy for iRBD from HCs.^41^

#### Trunk mobility/axial signs

Trunk mobility and axial signs were assessed and compared in three studies,^22,38,41^ which are summarized in Supplemental Table 7. One study used the UPDRS-III to measure axial signs, reporting that participants with iRBD had significantly more axial signs than HCs.^22^ Another study assessed trunk mobility during gait with wearable sensors, reporting that iRBD exhibited decreased trunk motion (reduced peak angular velocity and range of motion of the trunk) while walking at normal pace, fast pace, and while dual-tasking.^38^ Finally, another study used a bend, twist, and touch test to assess trunk mobility, which required participants to stand with their back to the wall before bending forward to touch a mark on the floor, then standing up and touching a mark on the wall behind them for 20 seconds. The total number of taps were counted. Individuals with iRBD performed worse on this task than HCs.^41^

#### Walking assessments

We subdivided walking assessments into clinical assessments (e.g., TUG, or other timed walk tests), and quantitative walking assessments (e.g., using pressure sensor walkways, or wearable sensors). All studies are summarized in Supplemental Table 8.

Twelve studies conducted clinical assessments of walking.^18,23,35,37,41,43,44,46,49,51–53^ Ten of these studies used the TUG.^18,23,37,41,43,44,49,51–53^ Of the studies that assessed TUG duration, seven reported that participants with iRBD took longer compared to HCs.^18,23,43,44,49,52,53^ Two studies did not find a difference in TUG duration.^41,51^ Additionally, two studies reported the TUG had poor discrimination accuracy for iRBD and HCs.^37,41^

Viteckova et al. had participants complete the TUG on a pressure sensor walkway under single-task, as well as cognitive (serially subtracting by 3s from 100) and motor (carrying a glass of water) dual-task conditions. They assessed gait characteristics in the pre-turn segment of the TUG, and did not find any group differences between iRBD and HCs in any gait outcomes.^48^ They did not compare TUG duration. Zatti et al. measured TUG duration, as well as characteristics of turning in the TUG using wearable sensors. They found that participants with iRBD exhibited decreased step time before the turn, as well as decreased peak and mean angular velocity when completing the TUG at their fastest pace.^51^

The remaining two studies that used clinical walking assessments used a 5-m walking test and a 10-m walking test (with single and dual-task conditions).^35,46^ The first study reported that participants with iRBD took longer to complete the 5-m walking test with less steps.^35^ The second study found that participants with iRBD took longer to complete the 10-m walking test only when dual-tasking.^46^

Eleven studies completed a quantitative gait assessment using either pressure sensor walkways, wearable sensors, or a smartphone.^16,17,22–26,38,39,48,51^ One of these studies focused on gait initiation, reporting deficits in anticipatory postural adjustments, the posterior shift of the center of pressure during the propulsive phase of gait initiation.^16^

Four studies measured gait outcomes during single-task overground walking, either on a pressure sensor-walkway^24,39^ or with wearable sensors.^22,38^ Two studies assessed gait with a pressure-sensor walkway.^24^^39^ The first study did not find differences when walking at a normal pace, however, step length asymmetry was greater in iRBD compared to HCs in fast-paced walking.^24^ The second study found that a diagnosis of probable RBD, based on the Mayo Sleep Questionnaire, was associated with reduced cadence, velocity, and stride length, and increased stride time variability, swing time variability, and double support time.^39^ Cochen de Cock et al. found that gait outcomes measured using six wearable sensors discriminated iRBD from HCs with excellent accuracy, particularly range of motion and asymmetry in the limbs, peak swing velocity and asymmetry in the limbs, trunk range of motion, trunk peak velocity, and gait phase difference and cadence.^22^ Ma et al. also measured gait using six wearable sensors, reporting that participants with iRBD exhibited decreased trunk motion (reduced peak angular velocity and range of motion) while walking and increased step time before turning compared to HCs.^38^ Therefore, changes in gait that have been reported in iRBD compared to HCs under single-task conditions included decreased velocity and stride length, increased step time, decreased trunk motion (reduced peak angular velocity and range of motion), decreased cadence, increased swing time, and increased variability in swing time, stride time and double support time.

Five studies used dual-tasking with a quantitative gait assessment.^22,24,25,38,48^ Dual-tasking revealed additional subtle gait deficits in one other study, namely increased step width variability.^24^ One study found decreased trunk motion (reduced peak angular velocity and range of motion), which was also seen in single-task walking.^38^ One study did not find impaired gait under dual-tasking conditions,^48^ and one study reported that dual-tasking did not improve discrimination accuracy of gait for iRBD and HCs.^22^ It is worth noting that these studies used an easier secondary cognitive task (subtracting by 3s) compared to the secondary cognitive tasks used in the studies that did detect gait differences when dual-tasking (e.g., subtracting by 7 s, or the VR Stroop task).^22,24,48^

Ehgoetz Martens et al. utilized a virtual-reality (VR) gait paradigm while participants laid supine inside a magnetic resonance imaging (MRI) machine and navigated a virtual environment using foot pedals. In the environment, participants navigated an environment under a single-task condition, and a dual-task condition that was simple (reacting to a simple “STOP” or “WALK” cue) or complex (a Stroop task), while step time and step time variability were measured from the foot pedals. HCs increased their step time in response to the complex dual-task, but individuals with iRBD did not.^25^

In another study, Ehgoetz Martens at al utilized a similar VR gait paradigm, but in this environment, participants navigated through wide and narrow doorways, Individuals with iRBD had longer step time and exhibited a greater increase in step time in response to HCs when navigating narrow doorways. They also had increased step time variability when navigating narrow doorways than HCs.^26^

Finally, Del Din et al., used a free-living gait protocol whereby gait characteristics were measured remotely with wearable sensors over a seven-day period, and they found that participants with iRBD walked slower with decreased gait velocity variability and reduced cadence (increased step time, swing time, and stance time) compared to HCs.^23^ Gait characteristics significantly discriminated iRBD from HCs, with swing time demonstrating the strongest accuracy.^23^ iRBD also had shorter ambulatory bouts on average and a greater ratio of short ambulatory bouts to long bouts (i.e., decreased alpha).^23^

One study reported that gait characteristics have very high accuracy at discriminating iRBD from HCs, particularly characteristics that represented limb range of motion and asymmetry, limb peak swing velocity and asymmetry, trunk range of motion, trunk peak velocity, and gait phase difference and cadence.^22^ On the other hand, Del Din et al found that micro gait measures of pace, variability and rhythm collected from free-living gait distinguished individuals with iRBD from HCs, with swing time as the best discriminator.^23^ In one study that used a smartphone to assess gait, gait features were among the most salient features that could discriminate iRBD from HCs, although they did not contribute the most, compared to other motor features.^17^

#### Falls

Cross-sectionally, one study compared falls rate, which was measured by asking participants about the number of falls they had in the past 6 months, reporting it was greater in iRBD than HCs.^23^

##### Multivariate analyses

Few of the studies also included analysis of the accuracy of a composite motor score or a motor battery at distinguishing iRBD from healthy controls.^37,41,46^ The first study calculated and validated a composite motor score from the scores of the UPDRS-III, TUG, and Purdue Pegboard test.^37^ Their results demonstrated that the composite score had greater discrimination accuracy for iRBD from controls than the TUG and Purdue Pegboard test, however the UPDRS-III score alone was more accurate.^37^

Two other studies conducted multivariate logistic regressions to assess the accuracy of a combined motor test battery.^41,46^ Nisser et al. included the falling stick test, the bend, twist and touch test, and the backwards line walking task in their model (as they significantly different between groups), but found that the combined model did not increase diagnostic accuracy.^41^ Additionally the falling stick test was the only significant predictor of group status in the combined model.^41^

Finally, Simonet et al. assessed the accuracy of a combined model that included their keyboard tapping test outcomes (BRAIN and DFT), change in 10-m walking time between single and dual task conditions, and variability in finger tapping amplitude during finger-tapping while dual-tasking.^46^ This combined model demonstrated greater accuracy, sensitivity and specificity at distinguishing iRBD from HCs than the UPDRS-III alone.^46^

### Longitudinal results

Table 2 also summarizes longitudinal studies included in this review in terms of authors, year of publication, country, inclusion/exclusion criteria, method of iRBD diagnosis, motor assessment methods and outcomes, and main findings. Note that if a study had a longitudinal design but motor function was only investigated at baseline or a single time point, we summarized this result under cross-sectional results as any conclusions from these studies are about cross-sectional differences. Supplemental tables also summarize longitudinal result by motor assessment method.

#### UPDRS

The 1987 version of the UPDRS was assessed over time in iRBD by one study in this review.^45^ The results demonstrated that this score deviated from normal approximately 4.5 years from phenoconversion. Researchers also divided the test by cardinal manifestations and location, finding that voice and face akinesia were the first to deviate, followed by rigidity, gait, limb bradykinesia and tremor.^45^

UPDRS-II was assessed longitudinally in four studies in this review. All four studies showed that UPDRS-II scores increase over time in iRBD.^27,33,34,50^ One of the longitudinal studies in this review estimated that UPDRS-II scores deviated from normal 9.3 years before phenoconversion and detected significant differences from HCs three years before.^27^

UPDRS-III was assessed longitudinally in eight studies in this review.^19,27,31–34,47,50^ Six of these studies demonstrated that UPDRS-III scores increase over time in iRBD,^27,32,33,35,47,50^ while two studies did not find an effect of time on UPDRS-III scores.^19,31^ Of the two studies that did not find a difference, one reported a mean follow-up duration of less than two years, which was the shortest of the eight studies, and the other had the smallest sample size.^19,31^

One longitudinal study reported that UPDRS-III scores in iRBD deviated from scores for healthy controls 5.6 years prior to phenoconversion. In this study, the UPDRS-II and III exhibited the highest accuracy at discriminating participants with iRBD who phenoconverted from HCs at phenoconversion, however these measures did not remain sensitive six years before phenoconversion (<50%).^27^

These studies are summarized in Supplemental Table 10.

#### Tapping assessments

Two studies measured the alternate tap test longitudinally in iRBD.^27,45^ They are summarized in Supplemental Table 11. One study found that deficits in the alternate tap test were the earliest motor deficits in iRBD, estimated to deviate from normal 12.9 years before phenoconversion and significantly differing from HCs at 6 years before phenoconversion.^27^ This study additionally found that the alternate tap test demonstrated excellent accuracy at phenoconversion and remained sensitive (>55%) up to 6 years before phenoconversion in discriminating between iRBD and HCs.^27^ In line with this, the alternate tap test was also the earliest motor outcome to deviate from normal (8 years prior to phenoconversion) in the second study, showed the greatest degree of progression over time, and was the most accurate and sensitive outcome at distinguishing iRBD from HCs at 3 years before phenoconversion.^45^

#### Purdue pegboard

The Purdue Pegboard test was measured longitudinally in iRBD by three studies in this review.^27,33,45^ These studies are summarized in Supplemental Table 12. All studies demonstrated that Purdue Pegboard performance worsens over time in iRBD.^27,33,45^ One study estimated that scores deviated from normal approximately 8 years before phenoconversion, while the other reported deviation from normal at 7.5 years prior to phenoconversion, with significant differences at four years prior to phenoconversion.^27,45^

#### Walking assessments

Three studies measured TUG performance longitudinally.^27,33,45^ They are summarized in Supplemental Table 13. All three studies demonstrated that TUG performance worsens over time in iRBD.^27,33,45^ However, one study noted that compared to the Purdue Pegboard and UPDRS-II and -III, the TUG had the smallest degree of progression in iRBD.^33^ Two of the studies estimated that TUG performance in iRBD deviated from HCs 6.5 and 6.3 years prior to phenoconversion.^27,45^

#### Falls

One longitudinal study assessed fall risk over one year between people with pRBD (diagnosed using the REM Sleep Behavior Disorder Questionnaire-Hong Kong) and without pRBD, reporting a 2.57-fold increase in risk of falling compared to HCs, after adjusting for age and sex. This remained significant after adjusting for several demographic covariates, medical history, lifestyle factors, and previous history of falls (See Table 2 for full list of covariates), although the odds ratio was reduced to 1.57.^29^

### Results of quality assessment

**Figure 2.**
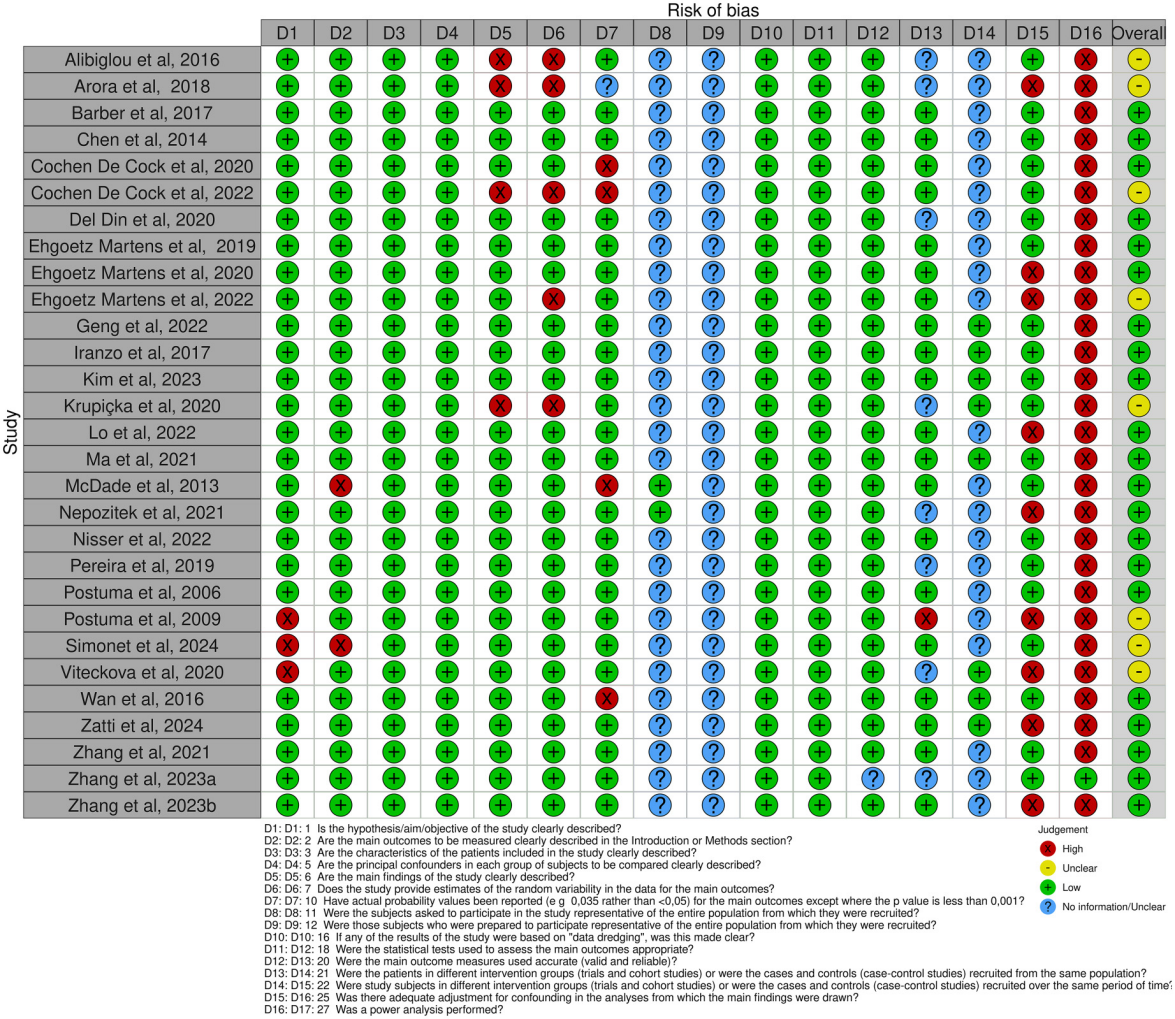
Summary of risk of bias assessment for cross-sectional studies, conducted using a modified Downs and Black checklist.

**Figure 3.**
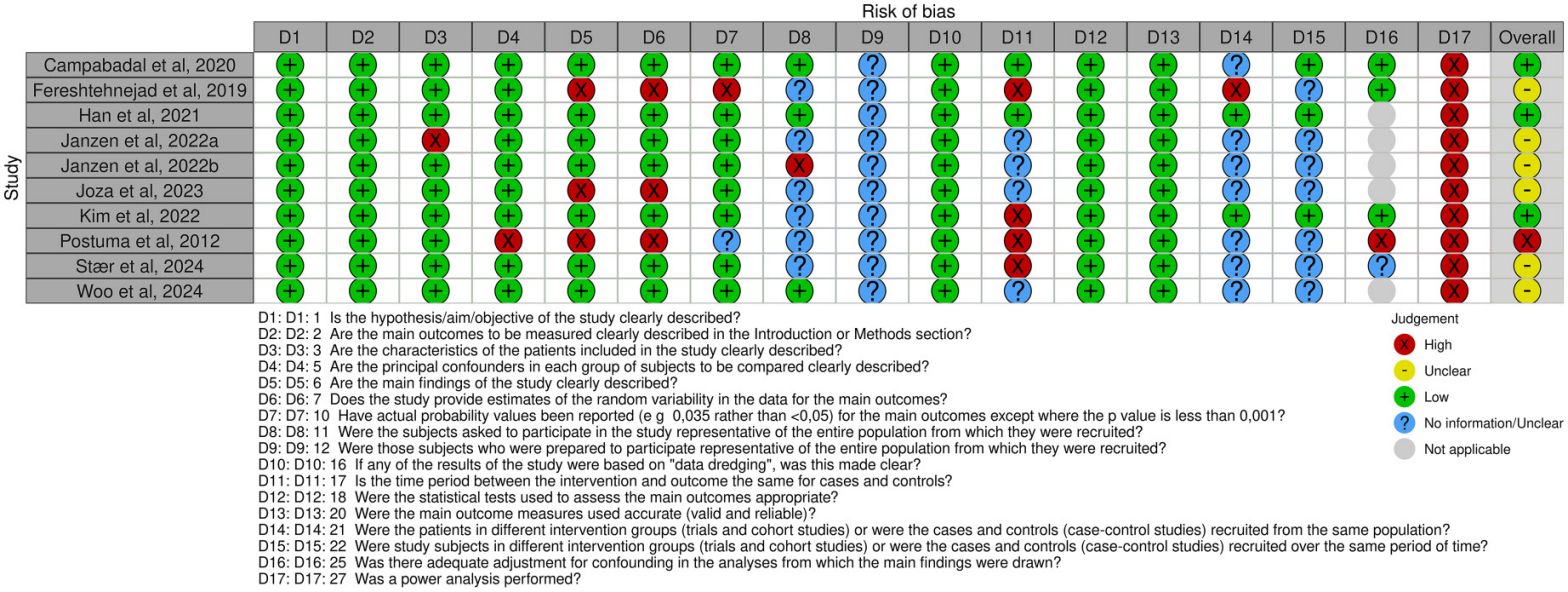
Summary of risk of bias assessment for longitudinal studies, conducted using a modified Downs and Black checklist.

The results of the quality assessment for each study are demonstrated in Figures 2 and 3, for cross-sectional and longitudinal findings, respectively. The intraclass correlation coefficient was calculated to be 0.6 (95% CI: 0.549, 0.647, p < 0.001), indicating moderate inter-rater reliability.

Of the 29 cross-sectional studies in this review, 21 studies had low risk of bias and eight had medium risk of bias. Scores ranged from 68% to 94% for the 21 studies with low risk of bias, and 44% to 63% for the eight studies with medium risk of bias. Of the ten longitudinal studies, three had low risk of bias, six had medium risk of bias, and one had high risk of bias. Scores ranged from 70% to 81% for the studies with low risk of bias, and 41% to 56% for the studies with medium risk of bias. The study with high risk of bias scored 29%.

Prominent deficits in reporting included sample size justifications through power calculations, describing sampling methods, as well as proper identification of when and from where participants were recruited.^11^ Longitudinal studies also often only followed the patient population (iRBD group) over time, and not the healthy controls. Item 12 of the checklist, which probes the representativeness of the study sample to the source population, was unclear for all the studies in this review as it required researchers validate that distribution of confounding variables was the same in the study's sample and the source population, which was not reported by any of the included studies.^11^

## Discussion

### Interpretation of findings

In order to understand which motor features can distinguish individuals with iRBD from HCs and how motor function changes over time in iRBD, we identified 39 studies that compared motor function between iRBD and HCs, or in iRBD over time. We characterized these studies by the type of motor assessment conducted. In this section, we will interpret our findings and discuss clinical implications of this research, risk of bias in the included studies, and finally future directions.

Overall, the results support the presence of subtle motor impairment in iRBD, even when parkinsonian signs are absent or very mild. Similarly, longitudinal studies showcase that motor function deteriorates over time in individuals with iRBD. These studies also demonstrate that the alternate tap test is one of the earliest motor deficits in iRBD.^27^ Most studies used clinical assessments, such as the UPDRS-III and the TUG, however, there is some inconsistency in the results, as some studies report they are different while other studies fail to detect a difference between participants with iRBD and HCs. Longitudinal studies, although fewer total, have more consistent results. This is not surprising, as cross-sectional studies may be affected by heterogeneity of iRBD so motor function may be variable across participants.

The UPDRS-II was assessed in fewer studies than the UPDRS-III and demonstrated more inconsistent results. The UPDRS-II focuses on activities of daily living and involves questions about daily tasks, self-care and social activities that are answered by the participant and/or their caregiver, whereas the UPDRS-III involves a detailed examination of motor features, including rigidity, tremor, bradykinesia, gait and postural instability, performed by the clinician. Individuals with iRBD more consistently performed worse on the UPDRS-III than the UPDRS-II, suggesting perhaps motor deficits are detected clinically but do not yet impact activities of daily living in participants with iRBD. On the other hand, in one study, UPDRS-II score was greater in iRBD than HCs, but not UPDRS-III.^44^ Fereshtehnejad et al. also estimated that UPDRS-II scores deviate from normal earlier than UPDRS-III scores in participants with iRBD who later phenoconverted.^27^ One possible explanation for this is because deficits in activities of daily living may also be impacted by non-motor symptoms of iRBD, which may not be detectable in motor functions evaluated by the UPDRS-III. Individuals with iRBD exhibit sleep dysfunction, but they also are at risk of mood disturbances, including anxiety and depression, which may impact daily tasks, and therefore UPDRS-II scores.^55^

We also found that in several studies that reported that clinical assessments failed to detect motor deficits, participants with iRBD did exhibit motor deficits detected through quantitative motor assessments. Furthermore, studies that assess the diagnostic value of these clinical assessments find that they are often not the most accurate or sensitive at distinguishing these groups, particularly when compared to other quantitative motor assessments, such as the alternate tap test, or finger-tapping assessments.^23,27,36,41^

In two longitudinal studies, the alternate tap test was found to detect the earliest motor manifestation in iRBD, and the most accurate at distinguishing iRBD individuals who phenoconverted from HCs.^27,45^ The alternate tap test also remained sensitive up to six years before phenoconversion, while other clinical tests, namely the UPDRS-II and -III did not.^27^ Similarly, the UPDRS finger-tapping performance was not different between iRBD and HCs in one study, but motion-capture of finger-tapping revealed deficits in finger-tapping, particularly in the amplitude of finger taps.^36^ This research also found that the finger-tapping outcomes were more accurate at distinguishing iRBD from HCs.^36^ This disconnect between clinical rating and quantitative measurement was also reflected in one study that assessed the TUG using wearable sensors to probe at deficits in turning, whereby they found that performance on the TUG (i.e., duration, as it is assessed clinically) did not differ between iRBD and HCs, however, deficits in turning in iRBD could be detected by the sensors.^51^ Overall, this suggests that iRBD may exhibit subtle motor deficits that are not detected by clinical assessments, particularly ther UPDRS, potentially due to floor effects as they are designed for use in PD, and not in prodromal disease. The inconsistency in study results using clinical assessments may also be due to heterogeneity in disease severity and duration in the iRBD samples, whereby these tests may be better able to detect motor deficits when participants are close to phenoconversion, particularly to PD. This is further supported by the finding that the UPDRS-II and -III did not remain sensitive at six years before phenoconversion, compared to the alternate tap test.^27^ This finding demonstrates that clinical scales developed for characterizing PD may be less able to capture motor deficits than quantitative methods, like the alternate tap test. Furthermore, it suggests that disease duration is an important confounder for these findings, as proximity to phenoconversion will impact changes in motor function and accuracy of different tests.

Interestingly, rhythm impairments in iRBD were consistently reported in studies that used tapping and gait assessments. These studies showed that iRBD have impaired cadence and rhythm during walking and arrhythmic finger-tapping, as well as increased incoordination in keyboard tapping tasks.^21–23,46^ Similar deficits in rhythm production have been reported previously in PD patients and may be linked to impairment in basal-ganglia-cortical circuitry that is involved in rhythm and timing.^22,56,57^ Therefore, it is possible that motor dysfunction in rhythm may be an early manifestation of degeneration of the basal ganglia prior to onset of parkinsonism.

Impairments in gait variability and asymmetry were also reported in people with iRBD compared to HCs.^22^^,23–26^ To our knowledge, variability and asymmetry in finger-tapping behavior has not previously been investigated, but it would be of interest to explore if individuals with iRBD who have asymmetrical and variable gait exhibit similar deficits in their upper limb mobility or finger dexterity. Since motor asymmetry is a cardinal feature of PD, it may also be the case that asymmetrical impairment appears as iRBD duration and severity increases. Researchers have suggested that rhythm impairments precede motor impairment that is characteristic of PD, particularly asymmetry.^21,22^ It is also a possibility that asymmetrical impairment in iRBD precedes PD phenoconversion, as opposed to other synucleinopathies. The pattern of motor deficits in iRBD leading to phenoconversion should be characterized in future studies in order to elucidate the significance of different types of motor deficits.

There is also a trend of an additional value in using dual-tasking with motor assessments, particularly because this may reveal motor impairment that is not apparent during single-task walking.^24,25,38^ For example, one study revealed that postural sway parameters increased in iRBD when performing a dual-task during quiet standing, compared to quiet standing alone.^24^ Consistent with this, two studies that assessed gait under single- and dual- task conditions found that dual-tasking uncovered impairments in gait that they did not detect when participants were performing motor tasks alone, without a dual-task.^24,25^ Similar findings were reported in finger-tapping.^46^ The type and difficulty of dual-tasking appears to be important, as studies using less difficult dual tasks (e.g., serially subtracting by 3s, instead of by 7s) did not report differences in motor outcomes, or an added predictive value.^22,48^ It is now understood that walking is not a fully automatic task, rather gait and balance control involve cognition and have attentional requirements. During dual-tasking, attentional resources are divided between gait control and the secondary task. This divided attention may lead to changes in walking behavior as resources available for motor control and coordination are compromised.^58^ On the other hand, it also appears that simply increasing task complexity can reveal impairments in motor function, for example, by walking at a fast pace or by walking through a narrow doorway.^24,26^ One study in particular reported that tasks that are more complex and may not be common in daily life (e.g., walking backwards) demonstrated the strongest accuracy at distinguishing iRBD from HCs when compared to clinical assessments or motor assessments that reflect common daily activities.^41^ Future research should explore the type and difficulty of secondary tasks in dual-task paradigms, or complexity of tasks that best uncover the subtle motor deficits in iRBD, and their potential utility as tools to identify RBD and estimate risk of phenoconversion. This work is particularly important considering that individuals with iRBD that experience cognitive deficits are at higher risk of developing DLB, which is characterized by deficits in cognition, particularly attention and executive function.^59^ Motor deficits being exacerbated during dual-tasking is important as it highlights that participants may be compensating for motor dysfunction using cognitive resources, which is impeded when a cognitive dual-task is introduced. Incorporating dual-tasking into motor assessments in clinical settings may be helpful for identifying early motor deficits in this group.

Another interesting finding in a few studies in this review is the potential for motor assessments in iRBD to be related to particular symptoms or subtypes of PD. For example, several studies found evidence of increased axial symptoms and reduced trunk mobility in iRBD compared to HCs, which suggests rigidity that is similar to what is seen in PD patients with the postural instability and gait disorder (PIGD) subtype of PD.^21,38,41^ Research has indicated individuals with RBD are more likely to develop the PIGD subtype. Similarly, other studies have found evidence of increased step time prior to turning, and gait initiation impairments that are similar to those seen in PD patients who experience freezing of gait (FOG).^16,38^ This may be an area of research that is worth investigating in future studies as this will help improve prediction of disease prognosis.

Finally, only a few studies investigated the accuracy of several motor tests or a motor battery at identifying iRBD from healthy controls. Although only one study found improved accuracy from a motor battery of tests, direct comparison of these results is difficult as different motor tests were combined in each study. Combining multiple motor measures can capture different aspects of motor impairment that are not easily captured in one motor test, highlighting the importance of considering a combination of motor assessments. Additionally, integrating motor assessments with non-motor markers, such as assessments of cognition, olfaction, or autonomic function, may further improve identification of iRBD and prediction of future phenoconversion. For example, Del Din et al. found that including the Sniffin' Sticks score, which is a measure of olfactory function, with their gait outcomes improved discrimination of iRBD from HCs, compared to the gait outcomes alone.^23^

### Strength of evidence

Based on the number of studies and consistency of results for each motor assessment, we organized studies from strongest evidence of motor deficits in iRBD to weakest evidence. We concluded there was strongest evidence of gait deficits, as walking was assessed in the greatest number of studies with relatively consistent findings. Clinical assessments such as the TUG, for example, did not always reveal motor deficits in iRBD and exhibited poor discrimination accuracy,^37,41^ however, studies using quantitative methods of evaluating gait, for example, wearable sensors, demonstrated more consistent findings of gait deficits in iRBD. In two studies, gait outcomes measured using wearable sensors demonstrated excellent discrimination accuracy between iRBD and HCs.^22,23^

We concluded that there was also strong evidence for tapping and balance assessments, as studies assessing these motor functions consistently reported motor deficits in iRBD compared to HCs. As mentioned previously, in one longitudinal study, the alternate tapping test was found to be the earliest motor deficit in iRBD, suggesting it may be an important screening tool for iRBD and monitoring proximity to phenoconversion.^27^ Studies assessing balance quantitatively using force platforms and wearable sensors have demonstrated that individuals with iRBD exhibit deficits in balance that were not demonstrated by studies that using clinical assessments of balance, namely the Flamingo test.^17,20,24,41^

There was relatively weak evidence for other clinical assessments, particularly the UPDRS-III. While in many studies it was shown that individuals with iRBD have greater scores, it discriminated iRBD from HCs with reduced accuracy compared to other quantitative motor assessments.^27,36,41^

Falls, tremor, and trunk mobility or axial signs were only measured in few studies, however, they consistently demonstrated greater deficits in iRBD than HCs in all studies that measured them. We concluded there is also relatively weak evidence for these deficits due to the small number of studies that have assessed them. Additional research is needed to confirm that these are motor deficits consistently found in iRBD compared to HCs.

Finally, we concluded there was the weakest evidence for the UPDRS-II, reaction time and Purdue pegboard for identifying motor deficits in iRBD, as the existing studies have inconsistent findings, with an equal number of studies reporting deficits are present and absent. Additional research is needed to clarify and explain these inconsistencies.

### Quality assessment

The results of our quality assessment revealed that most of the eligible studies had low risk of bias, while some had medium risk, and one had high risk. Overall, studies had good reporting of their aims, outcomes, and main findings. In the external validity section of the Downs and Black checklist, we consistently found that most studies did not adequately describe their sampling method or the source of the populations in the study, making it difficult to make conclusions about how well the sample in each study represented the source population.

Most studies were cross-sectional, limiting any conclusions that can be made about the progression of motor impairment in iRBD. One common source of risk of bias in the longitudinal studies included in this study is a different follow-up length between groups, whereby only the iRBD group had longitudinal follow-up, not the control group. Following both individuals with iRBD and HCs over time is important to distinguish changes in motor function due to aging, from those due to the neurodegenerative progression of iRBD. Most studies described and adjusted for potential confounders adequately, however, there were a few studies that reported differences in sex, or cognitive function, that were not adjusted for in their analyses. Few studies included RBD disease duration as a confounder, which is a limitation as this may affect results, considering longer disease durations may suggest greater proximity to phenoconversion, and therefore motor differences are more likely to be detected. Finally, some of the included studies did not assess cognitive function, which is important as this could potentially confound study findings, particularly if the subjects in the iRBD or HCs groups exhibit impaired cognition which may be indicative of latent neurodegeneration.

Finally, only one study adequately reported a sample size justification through a power calculation, which is important to allow readers to make conclusions about non-significant outcomes.

### Clinical implications

Clinically, the findings from this review suggest that clinical scales such as the UPDRS-III are inconsistent when it comes to distinguishing individuals with iRBD from HCs, potentially due to floor effects. On the other hand, quantitative motor assessments may provide more useful tools that can reveal subtle motor deficits in iRBD, prior to the onset of parkinsonian signs. Additionally, incorporating dual-tasking in motor assessments can aid in revealing motor impairment in this population. Quantitative motor assessments may therefore be useful as a first line of assessment for phenoconversion risk, as they are relatively cost-effective, non-invasive, and easy to implement. This can be followed with more specific screening, such as neuroimaging (e.g., dopamine transporter imaging) assess neurodegeneration and phenoconversion risk. It is worth noting that not all methods of motor assessment are inexpensive or easy to implement, for example using gait walkways or force plates to investigate gait and balance, would require additional costs and complex set up. This highlights an opportunity for future research to investigate methods to assess motor function in this population that are more accessible for clinicians.

The presence of motor deficits in iRBD prior to phenoconversion suggest motor function may be a helpful marker of phenoconversion risk; however, further research is needed to characterize changes in motor function over time in iRBD, and how well specific motor deficits predict phenoconversion risk.

Recently, a new biological staging system for PD has been proposed, which incorporates subtle motor deficits, which do not involve functional impairment, in stage 2B of the disease. This further underscores the importance of motor markers in the prodromal stage of the disease, before clinical symptoms of PD are present. This makes them potentially important outcomes in clinical trials for disease-modifying treatments to monitor disease progression in early stages of the disease and in iRBD.

### Future directions

Of the 39 studies in this review, we identified only ten studies that included longitudinal follow-up. One of our study aims was to characterize how motor deficits change over time in iRBD, however, due to the limited research, we are not able to make firm conclusions for this aim. It is clear that motor functions deteriorates over time in iRBD, but the existing longitudinal research has largely used clinical scales, most frequently the UPDRS-III, to measure motor functoin only. A variety of quantitative motor assessments should be included in future research to better capture the subtleties of motor progression in iRBD. Future studies should use repeated motor assessments in iRBD over time to uncover the pattern by which motor manifestations emerge as prodromal disease progresses, which will improve our ability to monitor phenoconversion risk. More longitudinal research is also needed to better assess how well motor assessments can predict future phenoconversion, as well as if they can distinguish PD and DLB converters. Recently, research by Postuma et al. highlighted that motor assessments such as the UPDRS-III, and quantitative motor assessments including the alternate tap test, Purdue Pegboard test, and TUG were predictive of phenoconversion in a large, multicenter cohort study of individuals with iRBD. This underscores the potential for motor deficits as biomarkers for identifying individuals at high risk of converting to overt α-synucleinopathies and highlights the importance of incorporating motor assessments in clinical practice and in the design of future neuroprotective trials in iRBD.^5^

Another possibility that is worth investigating in future research is whether these motor assessment outcomes can allow prediction of specific subtypes or symptoms of PD. As mentioned above, some studies identified rigidity in the trunk in iRBD, which may develop into the PIGD subtype of PD.^38,41^ Other studies identified impairments in gait and turning that are similar to impairments seen in PD patients who experience FOG.^16,38^ Longitudinal research is needed to confirm whether these motor deficits develop into these subtypes or symptoms of PD. This would also help improve our prediction of phenoconversion and disease prognosis, which can have important implications for early intervention strategies.

Furthermore, as mentioned previously, only few studies conducted multivariate analyses to investigate the accuracy of composite scores or motor batteries, as opposed to individual tests. This is an important future direction to help identify combinations of motor features that could be useful predictors of progression towards phenoconversion.

Finally, it is likely that the variability in results between studies can be explained by heterogeneity between iRBD samples, meaning it is possible that samples in one study where motor deficits are found are closer to phenoconversion than samples in studies that do not detect any motor deficits in the same or similar motor outcomes. Currently, there is no method to estimate proximity to phenoconversion in iRBD. Therefore, an important next step in iRBD research is the need to characterize heterogeneity in this population, particularly by estimating disease severity or proximity to phenoconversion. While the RBD Symptom Severity Scale is a novel tool that has been validated for characterizing severity of RBD symptoms, it is not clear how well this correlates to risk of phenoconversion, making it an important next step for future research.^60^ It is also important to note that because of the heterogeneity of iRBD, a comprehensive phenotyping approach is needed to better understand and predict phenoconversion risk. This should include not include only motor features, but also considerations of PSG findings, iRBD symptom duration, and other prodromal features, such as cognitive and autonomic function, neuroimaging, etc. This approach would allow for better risk estimation for phenoconversion, as well as predicting which specific synucleinopathy a given individual is at risk for.

It is also worth noting that motor deficits also exist in DLB, which indicates that subtle motor deficits detected in iRBD may still precede phenoconversion to DLB, and not PD alone. This may be another source of heterogeneity in iRBD samples, as they can convert to either PD or DLB. Future work should investigate whether motor deficits in iRBD can differentiate participants that will advance to PD and DLB, as well characterize the progression of these motor deficits to phenoconversion.

### Limitations

A key limitation of our review is the absence of a meta-analysis, which would have clarified consistency of results and magnitude of effect sizes between studies. To our knowledge, this is the first review to summarize existing literature. Because of this, we intentionally included a wide range of motor assessments used in previous studies. This broader inclusion allowed us to capture the diversity of motor features in iRBD but hindered our ability to conduct a meta-analysis due to the heterogeneity in study design and motor outcomes assessed in each study. This further highlights a need to standardize motor assessment methods across different studies to improve generalizability of findings.

Furthermore, while this review aimed to characterize motor features in individuals with iRBD, we acknowledge that other motor features, namely speech and facial expression, were not included. Although several studies have examined speech changes in iRBD, we opted to exclude them from our review as they rely on different assessment methods than motor assessments focused on gait or limb movements, which would have made direct comparisons of findings difficult. Given that speech and facial expression have potentially important relevance for early disease detection in iRBD, future research should continue to characterize these changes, as well as how changes in speech relate to broader motor deficits in iRBD.

## Conclusion

In conclusion, the existing literature supports the existence of subtle motor deficits in iRBD prior to phenoconversion, with the strongest evidence for walking, tapping, and balance deficits. There is inconsistency in the results of existing studies, which may be explained by heterogeneity in participants with iRBD. For this reason, an important next step is to develop a method of characterizing this heterogeneity and staging iRBD severity and proximity to phenoconversion. Longitudinal studies are needed to characterize the progression of motor impairment in iRBD to improve our understanding of prodromal disease progression and assess which motor assessments can best predict future phenoconversion. Gaining an understanding of which motor impairments can distinguish iRBD from HCs, and how motor function deteriorates in iRBD can reveal the utility of motor deficits as markers of disease progression and phenoconversion, which is particularly relevant for identifying useful biomarkers for clinical trials of neuroprotective therapies in iRBD.

## Supplemental Material

sj-docx-1-pkn-10.1177_1877718X251359225 - Supplemental material for Motor features that distinguish isolated REM sleep behavior disorder patients from healthy controls: A systematic review Supplemental material, sj-docx-1-pkn-10.1177_1877718X251359225 for Motor features that distinguish isolated REM sleep behavior disorder patients from healthy controls: A systematic review by Salma Elasfar, Hajr Hameed and Kaylena Ehgoetz Martens in Journal of Parkinson's Disease
