# Supplementary material for: Motor features that distinguish isolated REM sleep behavior disorder patients from healthy controls: A systematic review
Source: J Parkinsons Dis. 2025 Sep 1;15(7):1155–93. doi: 10.1177/1877718X251359225 (PMC13347522; doi:10.1177/1877718X251359225)
Supplement: sj-docx-1-pkn-10.1177_1877718X251359225 - Supplemental material for Motor features that distinguish isolated REM sleep behavior disorder patients from healthy controls: A systematic review [file sj-docx-1-pkn-10.1177_1877718X251359225.docx]

**Supplemental Material**

**Motor features that distinguish isolated REM sleep behavior disorder patients from healthy controls: A systematic review**

**Cross Sectional Differences in Motor Function between iRBD and HCs**

**Supplemental Table 1: Cross-sectional differences in UPDRS between iRBD and HCs**

| Study | Sample | Main findings |
| --- | --- | --- |
| UPDRS-II | | |
| Cochen De Cock et al., 2020^21^ | iRBD: n = 21 (19% F); mean age = 68.7±6.9  HCs: n = 38 (18.4% F); mean age = 69.1±7.2 | UPDRS-II score was not different between groups. |
| Cochen De Cock et al., 2022^22^ | iRBD: n = 21 (19% F); mean age = 68.7±6.9  HCs: n = 21 (19% F); mean age = 69.8±6.8 | UPDRS-II score was not different between groups. |
| Nepozitek et al., 2021^40^ | iRBD: n = 34 (14.7% F); mean age = 67.7±7.2; disease duration = 8.0±6.6; MoCA = 23.4±3.4  HCs: n = 33 (30.3% F); mean age = 61.5±8.2; MoCA = 25.7±2.3 | iRBD group had a greater UPDRS-II score than HCs at baseline. |
| Postuma et al., 2009^44^ | iRBD: n = 68 (22% F); mean age^Φ^ = 68.0 (44-93)  HCs: n = 36 (22.2% F); mean age^Φ^ = 65.8 (46-87) | iRBD group had a greater UPDRS-II score than HCs. |
| UPDRS-III | | |
| Alibiglou et al., 2016^16^ | iRBD: n = 10 (40% F), mean age = 61.5±8.6  HCs: n = 10 (20% F), mean age = 62.7±11.5 | UPDRS-III score was not different between groups. |
| Barber et al., 2017^18^ | iRBD: n = 171 (11.7% F); mean age = 64.7±9.0  HCs: n = 296 (51% F); mean age = 64.9±10.2 | iRBD group had a greater UPDRS-III score than HCs. |
| Chen et al., 2014^19^ | iRBD: n = 24 (29.2% F), mean age = 65.37±8.50  HCs: n = 23 (30.4% F); mean age = 64.21±7.27 | UPDRS-III score was not different between groups. |
| Cochen De Cock et al., 2020^21^ | iRBD: n = 21 (19% F); mean age = 68.7±6.9  HCs: n = 38 (18.4% F); mean age = 69.1±7.2 | UPDRS-III score was not different between groups. |
| Cochen De Cock et al., 2022^22^ | iRBD: n = 21 (19% F); mean age = 68.7±6.9  HCs: n = 21 (19% F); mean age = 69.8±6.8 | UPDRS-III score was not different between groups. |
| Del Din et al., 2020^23^ | iRBD: n = 63 (7.9% F); mean age = 67.1±9.4  HCs: n = 34 (0% F); mean age = 67.3±10.1 | iRBD group had a greater UPDRS-III score than HCs. |
| Ehgoetz Martens et al., 2019^24^ | iRBD: n = 24 (25% F); mean age = 66.9±7.6  HCs: n = 24 (42.8% F); mean age = 67.4±10.1 | iRBD group had a greater UPDRS-III score than HCs. |
| Geng et al., 2022^28^ | iRBD: n = 21 (33.3% F), mean age = 61±10.68; education = 8.81±3.40; MMSE† = 27 (26, 28)  HCs: n = 22 (40.9% F), mean age = 60.27±7.60; education = 8.86±3.14; MMSE† = 28 (27, 28) | iRBD group had a greater UPDRS-III score than HCs at baseline. |
| Iranzo et al., 2017^29^ | iRBD: n = 20 (20% F); mean age = 72.9±8.6  HCs: n = 32 (28.1% F); mean age = 69.5±6.0 | iRBD group had a greater UPDRS III score than HCs. |
| Kim et al., 2023^35^ | iRBD: n = 21 (47.4% F); mean age = 68.7±7.56  HCs: n = 17 (52.6% F); mean age = 64.2±6.7 | iRBD group had a greater UPDRS III score than HCs. |
| Krupiçka et al., 2020^36^ | iRBD: n = 40 (12% F); mean age = 68.0±6.0  HCs: n = 25 (12% F); mean age = 66.0±7.0 | iRBD group had a greater UPDRS III score than HCs. |
| Lo et al., 2022^37^ | iRBD: n = 272 (12% F); mean age = 65.2±8.8  HCs: n = 316 (47% F); mean age = 64.8±10.1 | The UPDRS-III had good discrimination accuracy and the highest discriminatory power for distinguishing iRBD and controls. |
| Ma et al., 2021^38^ | iRBD: n = 31 (16.1% F); mean age^†^ = 69 (63, 73)  HCs: n = 20 (55% F); mean age^†^ = 70 (67, 73) | iRBD group had a greater UPDRS III score than HCs. |
| Nepozitek et al., 2021^40^ | iRBD: n = 34 (14.7% F); mean age = 67.7±7.2; disease duration = 8.0±6.6; MoCA = 23.4±3.4  HCs: n = 33 (30.3% F); mean age = 61.5±8.2; MoCA = 25.7±2.3 | iRBD group had a greater UPDRS III score than HCs at baseline. |
| Pereira et al., 2019^42^ | iRBD: n = 27 (18.5% F); mean age = 68.9±5.5; education = 12.7±5.2; MoCA = 25.3±4.5  HCs: n = 31 (35.5% F); mean age = 58.5±11.0; education = 16.5±3.1; MoCA = 28.3±1.2 | iRBD group had a greater UPDRS III score than HCs at baseline. |
| Postuma et al., 2006^43^ | iRBD: n = 25 (12% F); mean age^Φ^ = 69.2 (44-93)    HCs: n = 25 (12% F); mean age^Φ^ = 69.2 (46-87) | iRBD group had a greater UPDRS III score than HCs. |
| Postuma et al., 2009^44^ | iRBD: n = 68 (22% F); mean age^Φ^ = 68.0 (44-93)  HCs: n = 36 (22.2% F); mean age^Φ^ = 65.8 (46-87) | UPDRS-III score was not different between groups. |
| Simonet et al., 2023^46^ | iRBD: n = 33 (9.1% F); mean age = 68.66±8.07  HCs: n = 29 (13.9% F); mean age = 69.65±7.74 | iRBD group had a greater UPDRS III score than HCs. |
| Wan et al., 2016^49^ | iRBD: n = 41 (41.5% F); mean age = 67±8.9  HCs: n = 63 (47.6% F); mean age = 67.9±7.0 | iRBD group had a greater UPDRS III score than HCs. |
| Zhang et al., 2023^54^ | iRBD: n = 41 (41.5% F); mean age = 67±8.9  HCs: n = 63 (47.6% F); mean age = 67.9±7.0 | iRBD group had a greater UPDRS III score than HCs at baseline. |

Abbreviations: F = female; HCs = healthy controls; iRBD = isolated REM sleep behavior disorder; UPDRS = Unified Parkinson’s Disease Rating Scale.

Participant characteristics data presented as mean ± standard deviation otherwise unless stated below:
^†^ Data is presented as median (upper quartile, lower quartile)

^Φ^ Data is presented as mean (range)

**Supplemental Table 2: Cross-sectional differences in tremor between iRBD and HCs**

| Study | Sample | Assessments | Main findings |
| --- | --- | --- | --- |
| Arora et al., 2018^17^ | iRBD: n = 104 (12% F); mean age = 64.5±9.4  HCs: n = 84 (33% F); mean age = 66.3±9.1 | Sit upright, hold phone in your tremor dominant hand and rest it lightly in your lap close your eyes and count backward from 100 (rest tremor), or with the arm outstretched in front of you (postural tremor). | Postural tremor and rest tremor were both among the most salient features that could discriminate iRBD from HCs, with postural tremor being the motor feature that contributed the most. |
| Cochen De Cock et al., 2020^21^ | iRBD: n = 21 (19% F); mean age = 68.7±6.9  HCs: n = 38 (18.4% F); mean age = 69.1±7.2 | Tremor measured by the UPDRS-III | The iRBD group had significantly more tremor than HCs. |
| Cochen De Cock et al., 2022^22^ | iRBD: n = 21 (19% F); mean age = 68.7±6.9  HCs: n = 21 (19% F); mean age = 69.8±6.8 | Tremor measured by the UPDRS-III | The iRBD group had significantly more tremor than HCs. |

Abbreviations: F = female; HCs = healthy controls; iRBD = isolated REM sleep behavior disorder

Participant characteristics data presented as mean ± standard deviation.

**Supplemental Table 3: Cross-sectional differences in tapping assessments between iRBD and HCs**

| Study | Sample | Assessments | Main findings |
| --- | --- | --- | --- |
| Clinical Assessments (e.g., Alternate tap test, Finger tapping item on UPDRS) | | | |
| Kim et al., 2023^35^ | iRBD: n = 21 (47.4% F); mean age = 68.7±7.56  HCs: n = 17 (52.6% F); mean age = 64.2±6.7 | Finger-tapping assessed per UPDRS instructions | Finger-tapping capacity was not different between iRBD and HCs. |
| Krupiçka et al., 2020^36^ | iRBD: n = 40 (12% F); mean age = 68.0±6.0  HCs: n = 25 (12% F); mean age = 66.0±7.0 | Finger-tapping measured by the UPDRS | Finger-tapping item subscore was not different between iRBD and HCs |
| Nisser et al., 2022^41^ | iRBD: n = 19 (31.6% F); mean age* = 70.6 [68.7, 72.5]  HCs: n = 20 (70% F); mean age* = 64.4 [60, 68.7] | Alternate tap test | Alternate-tapping test performance did not differ between groups. |
| Postuma et al., 2006^43^ | iRBD: n = 25 (12% F); mean age^Φ^ = 69.2 (44-93)    HCs: n = 25 (12% F); mean age^Φ^ = 69.2 (46-87) | Alternate tap test | iRBD group performed worse on the alternate tap test than HCs. |
| Postuma et al., 2009^44^ | iRBD: n = 68 (22% F); mean age^Φ^ = 68.0 (44-93)  HCs: n = 36 (22.2% F); mean age^Φ^ = 65.8 (46-87) | Alternate tap test | iRBD group performed worse on the alternate tap test than HCs. |
| Wan et al., 2016^49^ | iRBD: n = 41 (41.5% F); mean age = 67±8.9  HCs: n = 63 (47.6% F); mean age = 67.9±7.0 | Alternate tap test | iRBD group performed worse on the alternate tap test than HCs. |
| Zhang et al., 2021^52^ | iRBD: n = 21 (28.5% F), mean age = 65.3±7.0  HCs: n = 28 (43.7% F), mean age = 62.9±5.1 | Alternate tap test | iRBD group performed worse on the alternate tap test than HCs. |
| Zhang et al., 2023^53^ | iRBD converters: : n = 21 (38.1% F); mean age = 66.42±5.71  iRBD non-converters: n = 24 (12.5% F); mean age = 64.5±5.12  HCs: n = 25 (24.0% F); mean age = 64.0±3.79 | Alternate tap test (score transformed to z-scores based on mean and standard deviation of HCs) | Alternate tap test performance did not differ between groups. |
| Quantitative finger-tapping assessment (e.g., Motion Capture, Keyboard tapping) | | | |
| Arora et al., 2018^17^ | iRBD: n = 104 (12% F); mean age = 64.5±9.4  HCs: n = 84 (33% F); mean age = 66.3±9.1 | Tap smartphone screen alternately keeping regular rhythm. | Finger-tapping task outcomes were among the most salient features that distinguished iRBD from HCs, but they did not contribute the most. |
| Cochen De Cock et al., 2020^21^ | iRBD: n = 21 (19% F); mean age = 68.7±6.9  HCs: n = 38 (18.4% F); mean age = 69.1±7.2 | Unpaced finger tapping test: Participants asked to tap for 60 seconds at most comfortable rate. Paced finger tapping test: Participants tapped with 1) sound of metronome and 2) to the beat of short musical excerpts. | Participants with iRBD tapped faster than controls in the unpaced tapping tests and tapped with more variability than controls in both tests. |
| Krupiçka et al., 2020^36^ | iRBD: n = 40 (12% F); mean age = 68.0±6.0  HCs: n = 25 (12% F); mean age = 66.0±7.0 | Finger-tapping test (as per UPDRS instructions) captured by contactless 3D motion capture system | iRBD exhibited greater decrement in amplitude of finger-tapping compared to HCs. |
| Simonet et al., 2023^46^ | iRBD: n = 33 (9.1% F); mean age = 68.66±8.07  HCs: n = 29 (13.9% F); mean age = 69.65±7.74 | BRadykinesia Akinesia INcorrdination test (BRAIN): alternate tapping of the ‘s’ and ‘;’ keys with the index finger  Distal Finger Tapping test (DFT): repeated tapping of down arrow key with left index finger whilst depressing the left arrow key with left middle finger  Finger tapping test assessed using Slow-Motion Analysis of Repetitive Tapping (SMART) under single-task and dual task (listing months of the year in reverse order, subtracting from 100 by 3s) conditions | iRBD performed worse on both keyboard tapping tests (less keystrokes, increased dwell time on keys and incoordination).  There were no differences in the finger-tapping task under single-task conditions, however, iRBD exhibited slower, more erratic tapping with lower amplitude while dual-tasking compared to controls. |

Abbreviations: F = female; HCs = healthy controls; iRBD = isolated REM sleep behavior disorder

Participant characteristics data presented as mean ± standard deviation unless otherwise stated below:

^Φ^ Data is presented as mean (range)

**Supplemental Table 4: Cross-sectional differences in reaction time between iRBD and HCs**

| Study | Sample | Assessments | Main findings |
| --- | --- | --- | --- |
| Arora et al., 2018^17^ | iRBD: n = 104 (12% F); mean age = 64.5±9.4  HCs: n = 84 (33% F); mean age = 66.3±9.1 | Press and hold on-screen button as soon as it appears and release it as soon as it disappears. | Reaction time outcomes were not among the most salient features that could discriminate iRBD from HCs. |
| Nisser et al., 2022^41^ | iRBD: n = 19 (31.6% F); mean age* = 70.6 [68.7, 72.5]  HCs: n = 20 (70% F); mean age* = 64.4 [60, 68.7] | Falling stick test: Catch a sudden plunging ruler stick with each hand. Difference between initial position and final position measured. | iRBD participants performed worse on the falling stick test (caught the stick later) than HCs. The falling stick test demonstrated the best discrimination accuracy. |

Abbreviations: F = female; HCs = healthy controls; iRBD = isolated REM sleep behavior disorder

Participant characteristics data presented as mean ± standard deviation unless stated below:

* Data is presented as mean [lower range of 95% CI, upper range of 95% CI]

**Supplemental Table 5: Cross-sectional differences in the Purdue Pegboard test between iRBD and HCs**

| Study | Sample | Assessments | Main findings |
| --- | --- | --- | --- |
| Barber et al., 2017^18^ | iRBD: n = 171 (11.7% F); mean age = 64.7±9.0  HCs: n = 296 (51% F); mean age = 64.9±10.2 | Purdue pegboard test | There was no difference in performance on the Purdue Pegboard test between iRBD and HCs. |
| Del Din et al., 2020^23^ | iRBD: n = 63 (7.9% F); mean age = 67.1±9.4  HCs: n = 34 (0% F); mean age = 67.3±10.1 | Purdue pegboard test | iRBD participants performed worse on the Purdue Pegboard test than HCs. |
| Lo et al., 2022^37^ | iRBD: n = 272 (12% F); mean age = 65.2±8.8  HCs: n = 316 (47% F); mean age = 64.8±10.1 | Purdue pegboard test | Purdue pegboard test performance had poor discrimination accuracy for distinguishing iRBD from HCs. |
| Nisser et al., 2022^41^ | iRBD: n = 19 (31.6% F); mean age* = 70.6 [68.7, 72.5]  HCs: n = 20 (70% F); mean age* = 64.4 [60, 68.7] | Grooved Purdue pegboard test | There was no difference in performance on the Purdue Pegboard test between iRBD and HCs. |
| Postuma et al., 2006^43^ | iRBD: n = 25 (12% F); mean age^Φ^ = 69.2 (44-93)    HCs: n = 25 (12% F); mean age^Φ^ = 69.2 (46-87) | Purdue pegboard test | There was no difference in performance on the Purdue Pegboard test between iRBD and HCs. |
| Postuma et al., 2009^44^ | iRBD: n = 68 (22% F); mean age^Φ^ = 68.0 (44-93)  HCs: n = 36 (22.2% F); mean age^Φ^ = 65.8 (46-87) | Purdue pegboard test | iRBD group performed worse on the Purdue pegboard test than HCs. |
| Wan et al., 2016^49^ | iRBD: n = 41 (41.5% F); mean age = 67±8.9  HCs: n = 63 (47.6% F); mean age = 67.9±7.0 | Purdue pegboard test | iRBD performed worse on the Purdue pegboard test than HCs. |

Abbreviations: F = female; HCs = healthy controls; iRBD = isolated REM sleep behavior disorder

Participant characteristics data presented as mean ± standard deviation unless otherwise stated below:

^Φ^ Data is presented as mean (range)

**Supplemental Table 6: Cross-sectional differences in balance between iRBD and HCs**

| Study | Sample | Assessments | Main findings |
| --- | --- | --- | --- |
| Clinical Assessments (e.g., Flamingo test) | | | |
| Barber et al., 2017^18^ | iRBD: n = 171 (11.7% F); mean age = 64.7±9.0  HCs: n = 296 (51% F); mean age = 64.9±10.2 | Flamingo test (%) | iRBD performed worse on the Flamingo test than HCs. |
| Del Din et al., 2020^23^ | iRBD: n = 63 (7.9% F); mean age = 67.1±9.4  HCs: n = 34 (0% F); mean age = 67.3±10.1 | Flamingo test (s) | iRBD did not perform differently on the Flamingo test than HCs. |
| Zhang et al., 2021^52^ | iRBD: n = 21 (28.5% F), mean age = 65.3±7.0  HCs: n = 28 (43.7% F), mean age = 62.9±5.1 | Flamingo test (median) | iRBD did not perform differently on Flamingo test than HCs. |
| Quantitative balance assessment (e.g., instrumented walkways, force plates, etc.) | | | |
| Arora et al., 2018^17^ | iRBD: n = 104 (12% F); mean age = 64.5±9.4  HCs: n = 84 (33% F); mean age = 66.3±9.1 | Stand upright unaided for 30 s. Balance recorded using smartphone. | Balance outcomes were among the most salient features that distinguished iRBD from HCs, but they did not contribute the most. |
| Chen et al., 2014^20^ | iRBD: n = 24 (29.2% F), mean age = 65.37±8.50  HCs: n = 23 (30.4% F); mean age = 64.21±7.27 | Wearable inertial sensors at L4-L5 were used to measure postural sway parameters as participants stood in 5 conditions: (i) eyes open, (ii) eyes closed, (iii) eyes open with dual task (subtracting by 3s from 100), and (iv) eyes closed with dual task, and (v) tandem standing with eyes open | Postural sway parameters increased in iRBD compared to controls in the eyes closed with dual task and tandem standing with eyes open conditions.   All sway parameters except JERK and RMS in the ML direction were increased in iRBD compared to controls in the eyes open with dual-task condition. |
| Ehgoetz Martens et al., 2019^24^ | iRBD: n = 24 (25% F); mean age = 66.9±7.6  HCs: n = 24 (42.8% F); mean age = 67.4±10.1 | Standing on pressure sensor carpet in a comfortable stance and performing 2 quiet stance trials for 30 seconds (eyes open and eyes closed), followed by 2 single-leg stance trials (on each leg). | There was a trend for iRBD participants to exhibit increased anterior–posterior RMS during eyes-closed static balance, however, this did not remain significant after adjusting for multiple comparisons. |
| Nisser et al., 2022^41^ | iRBD: n = 19 (31.6% F); mean age* = 70.6 [68.7, 72.5]  HCs: n = 20 (70% F); mean age* = 64.4 [60, 68.7] | Static Balance: Total CoP excursion measured by force plate while participants stands in bipedal stance, right pedal stance, and left pedal stance. | There were no group differences in static balance, and none of the balance tests discriminated iRBD from HCs. |
| Dynamic Balance | | | |
| Nisser et al., 2022^41^ | iRBD: n = 19 (31.6% F); mean age* = 70.6 [68.7, 72.5]  HCs: n = 20 (70% F); mean age* = 64.4 [60, 68.7] | Dynamic Balance: Number of missteps counted while walking on 15- and 20- meter straight lines forwards and backwards. | iRBD group had more missteps during the backwards walk tests. The backwards line test showed good discrimination accuracy. |

Abbreviations: F = female; HCs = healthy controls; iRBD = isolated REM sleep behavior disorder; ML = medial-lateral; RMS = root mean square

Participant characteristics data presented as mean ± standard deviation unless otherwise stated below:

* Data is represented as mean [lower range of 95% CI, upper range of 95% CI]

**Supplemental Table 7: Cross-sectional differences in trunk/axial mobility between iRBD and HCs**

| Study | Sample | Assessments | Main findings |
| --- | --- | --- | --- |
| Cochen de Cock et al., 2022^21^ | iRBD: n = 21 (19% F); mean age = 68.7±6.9  HCs: n = 21 (19% F); mean age = 69.8±6.8 | Axial signs measured by the UPDRS-III | iRBD participants exhibited significantly more axial signs than HCs. |
| Ma et al., 2021^38^ | iRBD: n = 31 (16.1% F); mean age^†^ = 69 (63, 73)  HCs: n = 20 (55% F); mean age^†^ = 70 (67, 73) | Gait analysis with six wearable gyroscope and accelerometer sensors on wrists, ankles, anterior sternum, and lower back; under three conditions: usual pace, fastest pace, with a dual task (subtracting from 100 by 7s). | iRBD patients exhibited decreased trunk motion (reduced peak angular velocity and range of motion of trunk) while walking under all three conditions. |
| Nisser et al., 2022^41^ | iRBD: n = 19 (31.6% F); mean age* = 70.6 [68.7, 72.5]  HCs: n = 20 (70% F); mean age* = 64.4 [60, 68.7] | Bend, twist, and touch test: Standing with back to the wall, participant bends forward to touch a mark on the floor then back up to touch a mark on the wall behind them. Total number of taps in 20 seconds measured. | Participants with iRBD performed worse on the bend, twist, and touch test than HCs. The test showed good discrimination accuracy between iRBD and HCs. |

Abbreviations: F = female; HCs = healthy controls; iRBD = isolated REM sleep behavior disorder

Participant characteristics data presented as mean ± standard deviation unless otherwise stated below:
^†^ Data is presented as median (upper quartile, lower quartile)

**Supplemental Table 8: Cross-sectional differences in walking between iRBD and HCs**

| Study | Sample | Assessments | Main findings |
| --- | --- | --- | --- |
| Clinical Assessments (e.g., TUG or Walk tests) | | | |
| Barber et al., 2017^18^ | iRBD: n = 171 (11.7% F); mean age = 64.7±9.0  HCs: n = 296 (51% F); mean age = 64.9±10.2 | TUG (duration) | iRBD group took longer to complete the TUG than HCs. |
| Del Din et al., 2020^23^ | iRBD: n = 63 (7.9% F); mean age = 67.1±9.4  HCs: n = 34 (0% F); mean age = 67.3±10.1 | TUG (duration) | iRBD group took longer to complete the TUG than HCs. |
| Kim et al., 2023^35^ | iRBD: n = 21 (47.4% F); mean age = 68.7±7.56  HCs: n = 17 (52.6% F); mean age = 64.2±6.7 | 5-meter walk-test: Duration and number of steps measured. | iRBD group took longer to complete the 5-m walk, with less steps. |
| Lo et al., 2022^37^ | iRBD: n = 272 (12% F); mean age = 65.2±8.8  HCs: n = 316 (47% F); mean age = 64.8±10.1 | TUG (duration) | TUG performance had poor discrimination accuracy for distinguishing iRBD from HCs. |
| Nisser et al., 2022^41^ | iRBD: n = 19 (31.6% F); mean age* = 70.6 [68.7, 72.5]  HCs: n = 20 (70% F); mean age* = 64.4 [60, 68.7] | TUG (duration) | Groups did not perform differently on the TUG. TUG showed poor discrimination accuracy between groups. |
| Postuma et al., 2006^43^ | iRBD: n = 25 (12% F); mean age^Φ^ = 69.2 (44-93)    HCs: n = 25 (12% F); mean age^Φ^ = 69.2 (46-87) | TUG (duration) | iRBD group took longer to complete the TUG than HCs. |
| Postuma et al., 2009^44^ | iRBD: n = 68 (22% F); mean age^Φ^ = 68.0 (44-93)  HCs: n = 36 (22.2% F); mean age^Φ^ = 65.8 (46-87) | TUG (duration) | iRBD group took longer to complete the TUG than HCs. |
| Simonet et al., 2024^46^ | iRBD: n = 33 (9.1% F); mean age = 68.66±8.07  HCs: n = 29 (13.9% F); mean age = 69.65±7.74 | 10-meter walking test under single and dual task (listing months of the year in reverse order, subtracting from 100 by 3s) conditions | iRBD group took longer to complete the 10-m walking test while dual tasking compared to controls. |
| Wan et al., 2016^49^ | iRBD: n = 41 (41.5% F); mean age = 67±8.9  HCs: n = 63 (47.6% F); mean age = 67.9±7.0 | TUG (duration) | iRBD group performed worse than HCs on the TUG. |
| Zatti et al., 2024^51^ | iRBD: n = 23 (17% F); mean age = 72.0±6.0  HCs: n = 65 (60% F); mean age = 69.0±6.0 | TUG (duration) performed at normal pace and at fast pace. | TUG duration did not differ between iRBD and HCs at normal pace or fast pace. |
| Zhang et al., 2021^52^ | iRBD: n = 21 (28.5% F), mean age = 65.3±7.0  HCs: n = 28 (43.7% F), mean age = 62.9±5.1 | TUG (duration) | iRBD group took longer to complete the TUG than HCs. |
| Zhang et al., 2023^53^ | iRBD converters: : n = 21 (38.1% F); mean age = 66.42±5.71  iRBD non-converters: n = 24 (12.5% F); mean age = 64.5±5.12  HCs: n = 25 (24.0% F); mean age = 64.0±3.79 | TUG (duration – transformed to z-scores based on mean and standard deviation of HCs) | Both iRBD groups performed worse on the TUG than HCs. |
| Quantitative Gait assessments (e.g., instrumented walkways or wearable sensors) | | | |
| Alibiglou et al., 2016^16^ | iRBD: n = 10 (40% F), mean age = 61.5±8.6  HCs: n = 10 (20% F), mean age = 62.7±11.5 | Self-initiated gait stepping on 2 adjacent force platforms, which recorded CoP and GRF outcomes. EMG on TA. | iRBD group exhibited significant reductions in the posterior shift of CoP during the propulsive phase of gait initiation and in the duration of the initial EMG burst in TA in iRBD patients. |
| Arora et al., 2018^17^ | iRBD: n = 104 (12% F); mean age = 64.5±9.4  HCs: n = 84 (33% F); mean age = 66.3±9.1 | Walk 20 steps forward, turn around and return back to starting position. Gait recorded using smartphone. | Gait outcomes were among the most salient features that distinguished iRBD from HCs, but they did not contribute the most. |
| Cochen De Cock et al., 2022^22^ | iRBD: n = 21 (19% F); mean age = 68.7±6.9  HCs: n = 21 (19% F); mean age = 69.8±6.8 | 20-m walking test under single-task and dual-task (counting backwards from 100 by 1s, and by 3s) conditions while wearing 6 inertial measurement units on wrists, ankles, sternum and L5 positions | Gait outcomes distinguished iRBD from healthy controls with excellent accuracy, with the greatest contribution from range of motion and asymmetry, peak swing velocity and asymmetry, range of motion, peak velocity, and phase difference and cadence. The dual-task did not have any added value. |
| Del Din et al., 2020^23^ | iRBD: n = 63 (7.9% F); mean age = 67.1±9.4  HCs: n = 34 (0% F); mean age = 67.3±10.1 | Free-living gait protocol conducted with tri-axial accelerometers worn on L5 for 7 days. | Mean bout length was significantly lower, and alpha (ratio of short to long ambulatory bouts) was significantly greater in iRBD compared to HCs when looking at long bouts.  iRBD participants walked slower with less variable velocity, and lower cadence: increased step time, swing time, and stance time, in total bouts, and in long bouts (with the exception of step velocity variability) compared to HCs. These characteristics significantly  discriminated RBD, particularly swing time. |
| Ehgoetz Martens et al., 2019^24^ | iRBD: n = 24 (25% F); mean age = 66.9±7.6  HCs: n = 24 (42.8% F); mean age = 67.4±10.1 | Walking on a pressure sensor walkway under 5 conditions: normal pace, fastest pace, normal pace while counting backwards by 1s, normal pace while naming animals, normal pace while counting backwards by 7s. | All participants exhibited more impaired gait with a dual-task. iRBD exhibited increased step length asymmetry during fast-pace walking. Controls exhibited increased step width while dual tasking, whereas iRBD participants did not widen their step width but increased their step width variability significantly. |
| Ehgoetz Martens et al., 2020^25^ | iRBD: n = 30 (20% F); mean age = 66.7±7.2  HCs: n = 28 (50% F); mean age = 65.6±8.1 | 1) VR gait paradigm performed while lying in MRI machine, using foot pedals to navigate VR environment, with single-task and dual-task (cues presented were simple ("STOP" or "WALK") or complex (Stroop task)).  2) Overground walking on pressure sensor carpet (under single-task and dual-task (naming as many animals as possible) conditions. | 1) HCs increased their step time in response to the complex cognitive task in VR, while iRBD participants did not.  2) Step time variability increased in dual task (naming animals) condition but did not differ between groups. iRBD group exhibited longer step time during dual-task walking compared to healthy controls, but not single-task walking. |
| Ehgoetz Martens et al., 2022^26^ | iRBD: n = 23 (82.6% F); mean age = 66.9±7.2  HCs: n = 17 (52.9% F); mean age = 65.5±8.2 | VR gait paradigm performed while lying in MRI machine, using foot pedals to navigate VR environment, where participants navigated through wide and narrow doorways. | iRBD participants had longer step time overall. The iRBD group exhibited a more exaggerated increase in step time compared to HCs when navigating narrow doorways. The iRBD group had greater step time variability than HCs when navigating narrow doorways, but not wide doorways. |
| Ma et al., 2021^38^ | iRBD: n = 31 (16.1% F); mean age^†^ = 69 (63, 73)  HCs: n = 20 (55% F); mean age^†^ = 70 (67, 73) | Gait analysis with six wearable gyroscope and accelerometer sensors on wrists, ankles, anterior sternum, and lower back; under three conditions: usual pace, fastest pace, with a dual task (subtracting from 100 by 7s). | iRBD patients exhibited decreased trunk motion (reduced peak angular velocity and range of motion) walking in all conditions. They also had increased step time before turning compared to healthy controls in normal-paced and fast-paced walking, but not while dual-tasking. |
| McDade et al., 2013^39^ | iRBD: n = 42 (19% F); mean age^†^ = 79.0 (75.3, 84.1  HCs: n = 492 (30% F); mean age^†^ = 79.4 (75.9, 84.0) | Gait analyses were performed using a pressure sensor walkway, as participants walked at a normal pace. | pRBD group had decreased cadence, and increased swing time and variability in swing time and double support time. pRBD diagnosis was associated with reduced cadence, velocity, stride length, and increased stride time variability, swing time variability, and double support time. |
| Viteckova et al., 2020^48^ | iRBD: n = 67 (13.4% F); mean age = 66.22±8.39  HCs: n = 40 (15% F); mean age = 64.23±8.23 | Participants performed the TUG on a pressure sensor walkway under single task, motor dual task (carrying glass of water), and cognitive dual task (serial 3s, with no task prioritization) conditions | There were no significant group differences between groups on any of the gait variables (velocity, step length, step time, step width, gait cycle time, swing (%), stance (%), single support (%), double support (%), CV of step length, CV of step width, step length asymmetry, step time asymmetry) in any of the conditions. |
| Zatti et al., 2024^51^ | iRBD: n = 23 (17% F); mean age = 72.0±6.0  HCs: n = 65 (60% F); mean age = 69.0±6.0 | TUG turn characteristics measured using wearable sensors while participants performed TUG at normal pace and fast pace | iRBD participants exhibited longer turn duration and lower mean and peak angular velocities compared to HCs, but only during normal speed TUG. iRBD group experienced a greater increase in TUG speed between normal and fast-paced TUG than HCs. |

Abbreviations: CoP = center of pressure; EMG = electromyography; F = female; GRF = ground reaction forces; HCs = healthy controls; iRBD = isolated REM sleep behavior disorder; MRI = magnetic resonance imaging; TA = tibialis anterior; TUG = Timed Up and Go, VR = virtual reality.

Participant characteristics data presented as mean ± standard deviation unless otherwise stated below:
^†^ Data is presented as median (upper quartile, lower quartile)

* Data is represented as mean [95% CI]

^Φ^ Data is presented as mean (range)

**Supplemental Table 9: Cross-sectional differences in falls between iRBD and HCs**

| Study | Sample | Assessments | Main findings |
| --- | --- | --- | --- |
| Del Din et al., 2020^22^ | iRBD: n = 63 (7.9% F); mean age = 67.1±9.4  HCs: n = 34 (0% F); mean age = 67.3±10.1 | Falls rate: Participants asked to report the number of falls in the past 6 months | iRBD participants had a greater falls rate than HCs. |

Abbreviations: F = female; HCs = healthy controls; iRBD = isolated REM sleep behavior disorder; pRBD = probable REM sleep behavior disorder

Participant characteristics data presented as mean ± standard deviation.

**Longitudinal Changes in Motor Function in iRBD**

**Supplemental Table 10: Longitudinal changes in UPDRS in iRBD**

| Study | Sample | Main findings |
| --- | --- | --- |
| UPDRS (1987 Version) | | |
| Postuma et al.,  2012^45^ | iRBD: n = 20 (25% F); mean age = 70.5±6.9; time from iRBD diagnosis = 2.8±3.0; RBD symptom duration = 6.5±4.0 | Total UPDRS scores deviated from normal values approximately 4.5 years before phenoconversion. Specifically voice and face akinesia were the first to deviate, followed by rigidity, gait, limb bradykinesia and tremor. |
| UPDRS-II | | |
| Fereshtehnejad et al., 2019^27^ | iRBD (individuals who phenoconverted): 55 (30.9% F); mean age = 64.6±9.5 | UPDRS-II motor symptoms were estimated to deviate from normal 9.3 years before phenoconversion, with significant differences from HCs at 3 years before phenoconversion. |
| Joza et al., 2023^33^ | iRBD: n = 1160 (21.6% F); mean age = 68.5±7.0; time from iRBD diagnosis = 1.28±2.3; self-reported iRBD duration = 6.4±6.4 | After mean follow-up of 3.3 years, UPDRS-II showed clear progression over time in iRBD. |
| Kim et al., 2022^34^ | iRBD: n = 28 (43% F); mean age = 69.8±5.7; disease duration = 4.7±3.9; age at iRBD diagnosis = 65.1±7.1; MMSE = 27.5±2.2  HCs: n = 28 (64% F); mean age = 70.2±4.4; MMSE = 28.4±1.5 | UPDRS-II scores increased after a median follow-up of 5.1 years in the iRBD group. |
| Woo et al., 2024^50^ | iRBD with constipation: n = 29 (41.4% F); mean age = 72.10±6.26; education = 7.64±4.49; MMSE = 26.10±3.22  iRBD without constipation: n = 24 (54.2% F); mean age = 67.58±7.77; education = 10.42±4.90; MMSE = 26.67±3.63 | After a mean follow-up of 4.08 years, UPDRS-II scores increased over time significantly in all iRBD groups. The yearly progression of these motor outcomes worsened over time at a greater rate in those with iRBD with constipation, than those with iRBD without constipation. |
| UPDRS-III | | |
| Campabadal et al., 2020^19^ | iRBD: n = 14 (21.4% F); mean age = 70.1±6.0; education = 10.1±5.1; disease duration = 4.5±3.4; age at iRBD onset = 65.6±7.5; MMSE = 27.9±1.7  HCs: n = 18 (61.1% F); mean age = 68.3±7.5; education = 10.9±4.2; MMSE = 29.4±1.0 | After a mean follow-up of 1.6 years, UPDRS-III scores did not change over time in the iRBD group. |
| Fereshtehnejad et al., 2019^27^ | iRBD (individuals who phenoconverted): 55 (30.9% F); mean age = 64.6±9.5 | UPDRS-III motor signs were estimated to deviate from normal 6.5 years before phenoconversion, with significant differences from HCs at 5 years before phenoconversion. |
| Janzen et al., 2021^30^ | iRBD with normal MIBG: n = 5 (20% F); mean age = 60.9±6.4; disease duration^†^ = 10.0 (7.6, 19.2); MoCA^†^ = 27.0 (24.5, 29.0)  iRBD with pathological MIBG: n = 12 (8% F); mean age = 63.5±5.3; disease duration^†^ = 7.8 (6.2, 9.6); MoCA^†^ = 27.0 (26.0, 28.0) | UPDRS-III did not change after approximately four-year follow-up in the normal MIBG or abnormal MIBG groups. |
| Janzen et al., 2021^31^ | iRBD with normal MIBG: n = 8 (25% F); mean age^†^ = 58.5 (53.8, 65.0); disease duration^†^ = 57.0 (12.5, 113.3) months; age at iRBD diagnosis^†^ = 58.5 (53.8, 65.0); MoCA^†^ = 26.5 (24.3, 28.5)  iRBD with pathological MIBG + normal FP-CIT: n = 9 (11.1% F); mean age^†^ = 66.0 (57.5, 71.0); disease duration^†^ = 55 (29.5, 151.0) months; age at iRBD diagnosis^†^ = 66.0 (57.5, 71.0); MoCA^†^ = 26.0 (24.0, 28.5)  iRBD with pathological MIBG + FP-CIT: n = 29 (10.3% F); mean age^†^ = 66.0 (62.5, 71.5); disease duration^†^ = 59.0 (25.5, 102.5) months; age at iRBD diagnosis^†^ = 59.0 (25.5, 102.5); MoCA^†^ = 27.0 (25.3, 28.8) | When participants were divided into normal and pathological MIBG groups, UPDRS-III significantly increased after follow-up in only those with iRBD and pathological MIBG.  When the pathological MIBG group was further subdivided based on whether FP-CIT was normal or abnormal, UPDRS-III significantly increased after follow-up in only those with iRBD and pathological MIBG and FP-CIT. |
| Joza et al., 2023^33^ | iRBD: n = 1160 (21.6% F); mean age = 68.5±7.0; time from iRBD diagnosis = 1.28±2.3; self-reported iRBD duration = 6.4±6.4 | After mean follow-up of 3.3 years, UPDRS-III showed the greatest degree of progression over time in iRBD. |
| Kim et al., 2022^34^ | iRBD: n = 28 (43% F); mean age = 69.8±5.7; disease duration = 4.7±3.9; age at iRBD diagnosis = 65.1±7.1; MMSE = 27.5±2.2  HCs: n = 28 (64% F); mean age = 70.2±4.4; MMSE = 28.4±1.5 | UPDRS-III scores increased after a median follow-up of 5.1 years in the iRBD group. |
| Stær et al., 2023^47^ | iRBD: n = 12 (20% F); mean age = 64.9±5.5; disease duration = 3.5±2.8; MMSE = 28.3±1.7; MoCA = 25.3±2.3  HCs: n = 9 (0% F); mean age = 64.3±6.9; MMSE = 29.6±0.7; MoCA = 26.8±2.7 | After a mean follow-up of 3.08 years, UPDRS-III scores increased from baseline in the iRBD participants. |
| Woo et al., 2024^50^ | iRBD with constipation: n = 29 (41.4% F); mean age = 72.10±6.26; education = 7.64±4.49; MMSE = 26.10±3.22  iRBD without constipation: n = 24 (54.2% F); mean age = 67.58±7.77; education = 10.42±4.90; MMSE = 26.67±3.63 | After a mean follow-up of 4.08 years, UPDRS-III scores increased over time significantly in all iRBD groups. The yearly progression of these motor outcomes worsened over time at a greater rate in those with iRBD with constipation, than those with iRBD without constipation. |

Abbreviations: F = female; HCs = healthy controls; iRBD = isolated REM sleep behavior disorder

Participant characteristics data presented as mean ± standard deviation otherwise unless stated below:
^†^ Data is presented as median (upper quartile, lower quartile)

**Supplemental Table 11: Longitudinal changes in tapping assessments in iRBD**

| Study | Sample | Assessments | Main findings |
| --- | --- | --- | --- |
| Clinical Assessments (e.g., Alternate tap test, Finger tapping item on UPDRS) | | | |
| Fereshtehnejad et al., 2019^27^ | iRBD (individuals who phenoconverted): 55 (30.9% F); mean age = 64.6±9.5 | Alternate tap test | Alternate tap test performance was estimated to deviate from normal 12.9 years before phenoconversion, with significant differences from HCs at 6 years before phenoconversion. |
| Postuma et al., 2012^45^ | n = 20 (25% F); mean age = 70.5±6.9; time from iRBD diagnosis = 2.8±3.0; RBD symptom duration = 6.5±4.0 | Alternate tap test | The alternate tap test deviated from normal approximately 8 years before phenoconversion. It showed the greatest degree of progression over time. The results of the ROC analysis showed that the alternate tap test was the most accurate and sensitive outcome for prediction of phenoconversion at 3 years before phenoconversion. |

Abbreviations: F = female; HCs = healthy controls; iRBD = isolated REM sleep behavior disorder

Participant characteristics data presented as mean ± standard deviation unless otherwise stated below:

**Supplemental Table 12: Longitudinal changes in Purdue Pegboard test in iRBD**

| Study | Sample | Assessments | Main findings |
| --- | --- | --- | --- |
| Fereshtehnejad et al., 2019^27^ | iRBD (individuals who phenoconverted): 55 (30.9% F); mean age = 64.6±9.5 | Purdue pegboard test | Purdue pegboard test performance was estimated to deviate from normal 7.5 years before phenoconversion, with significant differences from HCs at 4 years before phenoconversion. |
| Joza et al., 2023^33^ | iRBD: n = 1160 (21.6% F); mean age = 68.5±7.0; time from iRBD diagnosis = 1.28±2.3; self-reported iRBD duration = 6.4±6.4 | Purdue pegboard test | Purdue Pegboard performance showed clear progression over time in iRBD. It demonstrated the second greatest degree of change over time, following UPDRS-III. |
| Postuma et al., 2012^45^ | n = 20 (25% F); mean age = 70.5±6.9; time from iRBD diagnosis = 2.8±3.0; RBD symptom duration = 6.5±4.0 | Purdue pegboard test | The Purdue pegboard test deviated from normal approximately 8 years before phenoconversion. |

Abbreviations: F = female; HCs = healthy controls; iRBD = isolated REM sleep behavior disorder

Participant characteristics data presented as mean ± standard deviation unless otherwise stated below:

**Supplemental Table 13: Longitudinal changes in walking assessments in iRBD**

| Study | Sample | Assessments | Main findings |
| --- | --- | --- | --- |
| Clinical Assessments (e.g., TUG or Walk tests) | | | |
| Fereshtehnejad et al., 2019^26^ | iRBD (individuals who phenoconverted): 55 (30.9% F); mean age = 64.6±9.5 | TUG (duration) | TUG performance was estimated to deviate from normal 6.5 years before phenoconversion, with significant differences from HCs at 2 years before phenoconversion. |
| Joza et al., 2023^33^ | iRBD: n = 1160 (21.6% F); mean age = 68.5±7.0; time from iRBD diagnosis = 1.28±2.3; self-reported iRBD duration = 6.4±6.4 | TUG (duration) | TUG duration showed clear progression over time in iRBD, however, it demonstrated the lowest degree of change over time compared to other motor assessments (Purdue Pegboard, and UPDRS-II and -III) |
| Postuma et al., 2012^45^ | n = 20 (25% F); mean age = 70.5±6.9; time from iRBD diagnosis = 2.8±3.0; RBD symptom duration = 6.5±4.0 | TUG (duration) | TUG duration deviated approximately 6.3 years from phenoconversion. TUG was the most specific outcome at 3 years before phenoconversion, however it was not the most sensitive motor outcome for detecting iRBD. |

Abbreviations: F = female; HCs = healthy controls; iRBD = isolated REM sleep behavior disorder

Participant characteristics data presented as mean ± standard deviation unless otherwise stated below:

**Supplemental Table 14: Longitudinal associations between falls and RBD**

| Study | Sample | Assessments | Main findings |
| --- | --- | --- | --- |
| Han et al., 2016^27^ | All participants: n = 6891 (61.4% F), mean age = 71.4 | Risk of falls (OR) based on faller status | pRBD diagnosis was associated with a 2.57-fold increase in risk of falling than in elderly without pRBD, after adjusting for age and sex. |

Abbreviations: F = female; HCs = healthy controls; iRBD = isolated REM sleep behavior disorder

Participant characteristics data presented as mean ± standard deviation unless otherwise stated below:
